# The Copenhagen Triage Algorithm is non-inferior to a traditional triage algorithm: A cluster-randomized study

**DOI:** 10.1371/journal.pone.0211769

**Published:** 2019-02-04

**Authors:** Rasmus Bo Hasselbalch, Mia Pries-Heje, Martin Schultz, Louis Lind Plesner, Lisbet Ravn, Morten Lind, Rasmus Greibe, Birgitte Nybo Jensen, Thomas Høi-Hansen, Nicholas Carlson, Christian Torp-Pedersen, Lars S. Rasmussen, Kasper Iversen

**Affiliations:** 1 Department of Cardiology, Herlev-Gentofte Hospital, Copenhagen, Denmark; 2 Department of Emergency Medicine, Herlev-Gentofte Hospital, Copenhagen, Denmark; 3 Department of Cardiology, Bispebjerg Hospital, Copenhagen, Denmark; 4 Department of Emergency Medicine, Bispebjerg Hospital, Copenhagen, Denmark; 5 Department of Cardiology, Gentofte Hospital, Copenhagen, Denmark; 6 The Danish Heart Foundation, Copenhagen, Denmark; 7 Department of Health, Science and Technology, Aalborg University and Department of Cardiology and Epidemiology/Biostatistics, Aalborg University Hospital, Aalborg, Denmark; 8 Department of Anaesthesia, Center of Head and Orthopaedics, Rigshospitalet, University of Copenhagen, Copenhagen, Denmark; Public Library of Science, UNITED KINGDOM

## Abstract

**Introduction:**

Triage systems with limited room for clinical judgment are used by emergency departments (EDs) worldwide. The Copenhagen Triage Algorithm (CTA) is a simplified triage system with a clinical assessment.

**Methods:**

The trial was a non-inferiority, two-center cluster-randomized crossover study where CTA was compared to a local adaptation of Adaptive Process Triage (ADAPT). CTA involves initial categorization based on vital signs with a final modification based on clinical assessment by an ED nurse. We used 30-day mortality with a non-inferiority margin at 0.5%. Predictive performance was compared using Receiver Operator Characteristics.

**Results:**

We included 45,347 patient visits, 23,158 (51%) and 22,189 (49%) were triaged with CTA and ADAPT respectively with a 30-day mortality of 3.42% and 3.43% (P = 0.996) a difference of 0.01% (95% CI: -0.34 to 0.33), which met the non-inferiority criteria. Mortality at 48 hours was 0.62% vs. 0.71%, (P = 0.26) and 6.38% vs. 6.61%, (P = 0.32) at 90 days for CTA and ADAPT. CTA triaged at significantly lower urgency level (P<0.001) and was superior in predicting 30-day mortality, Area under the curve: 0.67 (95% CI 0.65–0.69) compared to 0.64 for ADAPT (95% CI 0.62–0.66) (P = 0.03). There were no significant differences in rate of admission to the intensive care unit, length of stay, waiting time nor rate of readmission within 30 or 90 days.

**Conclusion:**

A novel triage system based on vital signs and a clinical assessment by an ED nurse was non-inferior to a traditional triage algorithm by short term mortality, and superior in predicting 30-day mortality.

**Trial registration:**

Clinicaltrials.gov NCT02698319

## Introduction

### Background

Triage systems have been implemented in most emergency departments (ED) to minimize crowding and treatment delays that can result in adverse patient outcomes [1, 2]. The purpose of triage is to identify patients that need immediate treatment upon ED presentation and to provide general risk stratification to optimize resource allocation.

There is a variety of triage models in use worldwide mostly based on systematized flow-charts of symptoms and vital signs leaving limited room for clinical judgement [3, 4]. Typical triage systems involve a two-step procedure. First, a standardized set of vital signs (e.g. heart rate, blood pressure etc.) are measured and compared with predetermined limits set for each triage level. Then the ED nurse compares the patient’s chief complaint (e.g. chest pain) to a chart for that symptom listing a set of specific discriminators (e.g. severe pain) along with a corresponding triage level. The final level of the patient is determined by the most urgent of the two triage levels [3].

Traditional triage systems have been implemented without good evidence of a clinically beneficial effect or quality studies indicating their predictive ability [5], and since triage requires both time and resources, they could potentially be of more harm than benefit.

The Copenhagen Triage Algorithm is (CTA) a new triage system designed to simplify risk prediction and to reintroduce clinical judgment as the central part of triage [6]. Here we compare CTA with a traditional triage model in terms of risk prediction and patient outcomes, with a focus on 30-day mortality.

## Methods

### Study design and setting

This is a two-center, cluster-randomized, cross-over, non-inferiority trial using hospitals as the units of randomization and patients as the units of analysis, in which we compare CTA to a traditional triage system. Herlev Hospital and Bispebjerg Hospital have two equally sized EDs in the Capital Region of Denmark and was recruited to perform the study as they manage 70,000 and 85,000 annual patient visits, respectively [7, 8]. Both centers are 24-hour acute care hospitals offering broad medical, surgical, neurological, level-2 trauma, and ICU services. These hospitals were selected as representative of large scale EDs because they both have a high intake of unselected acute patients of all age classes across both medical and surgical specialties. We introduced CTA in “active” cluster periods, while “control” cluster periods employed the pre-existing triage system. A coin toss was used to decide the allocation of active and control clusters. We gathered baseline data from the chart made for each patient at admission. We collected data on contacts to the secondary health care system including admissions from The Danish National Patient Register, in which every resident of Denmark has a unique identifier that enables individual linkage of administrative registers [9], and we obtained vital status from the Central Personal Register, in which complete data of every death in Denmark is registered. Additional details regarding study design and rationale are described elsewhere [6]. This study was reviewed by the Regional Ethics Committee, which decided that no formal approval was needed according to Danish law.

### Selection of participants

We included all patients admitted through the EDs at either of the two participating centers during the study period. We excluded patients presenting in the ED with minor injuries (e.g. sprained ankle or minor cuts/abrasions) as they are triaged as blue (level 5) in both systems which typically precludes measurement of vital signs, as well as readmissions within 90 days of the index admission. This design led to an inclusion rate of close to 100%, as all acutely admitted patients were triaged and the two models were never in use at the same time at any center. However, we also excluded patients admitted directly to their respective departments, including patients younger than 16 years and gynecology and obstetrics patients, as well as patients with major trauma suspected pre-hospital who were admitted to a tertiary center in the region.

### Interventions

The Copenhagen Triage Algorithm (CTA) is a new triage system constructed from data collected from 12 000 ED patient visits at a large Danish hospital [10, 11].

The CTA solely consists of a quick score of vital signs followed by a basic clinical assessment by an ED nurse. The final triage level results from a score calculated after measurement of vital signs corresponding to a triage level of 1–4, that is then adjusted by the ED nurse, who can either up-triage (up to 2 steps) or down-triage (1 step) according to clinical assessment with no specific explanation needed. The development of the CTA has previously been described in detail [6].

A local adaption of the Adaptive Process Triage (ADAPT), triage model developed in Sweden in 2006 similar to most other well-known triage models [6, 10, 12, 13], was the standard triage model used in the study. It includes the presenting (or chief) complaint in addition to a list of vital signs with cut-off values corresponding to each triage level. These are both registered, and the final triage level is determined by the most urgent triage level assigned. The two triage systems are illustrated in S1 Fig.

Both triage systems identify five levels of urgency with colors (red, orange, yellow, green, and blue). The least urgent of these (the fifth level) was excluded from this study as it is defined as non-emergent patients (e.g. minor injuries) and includes no indication for measuring vital signs.

### Outcomes

The primary aim of the study was to assess whether CTA is non-inferior to ADAPT, as determined by patient outcomes. We also investigate differences in triage distributions and compare the predictive ability of the two algorithms for risk assessment. We define the primary endpoint as all-cause 30-day mortality, and secondary end-points include acute (48 hours) and long-term mortality (90 days) as well as rate of admission to the intensive care unit. Other secondary endpoints were designed to assess patient flow and resource utilization and included waiting time from triage to start of treatment, length of stay and readmission, as well as a post hoc analysis of how many patients left without being seen by a doctor, and in-hospital mortality. We assessed all endpoints in October 2016.

### Analysis

Based on earlier work, we estimated that 30-day mortality would be 4.2% at each center [10, 11] and the non-inferiority margin (Δ) was set to 0.5%. This required a sample size of at least 39,820 patient visits in total with a power of 80% and a two-sided confidence interval of 95%.

We evaluated time-to-event data using a semi-parametric procedure fitting a Cox proportional hazard model for marginal effects stratified by cluster. We used receiver operating characteristics (ROC) and area under the curve (AUC) statistics to compare the ability of each model to predict mortality, in these analyses the triage categories were viewed as numeric scores. We used the Youden index and calculated sensitivity, specificity, negative predictive value and positive predictive value for the prediction of 30-day mortality for each triage system. To assess calibration of the triage algorithms we internally validated the predictive abilities using a cross-validation approach using 1000 splits into training and validation set from which we calculated Brier scores. We computed a Forest plot encompassing sensitivity analyses to appraise the safety of CTA across differing demographics. Mortality endpoints were assessed by the intention-to-treat principle, while all statistical analyses regarding prognostic abilities were done per-protocol and excluded patients with no or erroneous triage. Mortality was also compared using Mantel-haenszel Chi square.

Continuous variables are described by median and interquartile range (IQR) and mean with standard deviation (SD). Categorical variables are described by number (n) and percentages (%). The baseline characteristics were compared using chi-square, Student’s *t*, and Wilcoxon tests. Student’s *t*-test was used to compare length of stay and chi-square or Fisher’s tests were used where appropriate to compare proportions.

We performed statistical programming in R version 3.3.3 [14] and SAS version 9.4 2002–2012 (SAS institute Inc., Cary, NC, USA).

### Ethics approval

The study was presented to the Regional Ethics Committee of Copenhagen (subcommittee D), who determined that no formal approval was needed in accordance with Danish law (H-4-2014-FSP), thus the need for consent was waived. The Danish Data Protection Agency (HEH-2014-118) and the Danish Health and Medicines Authority (3-3013-1119/2). The study is registered on clinicaltrials.gov (NCT02698319).

### Trial registration

Our trial was unfortunately registered after initiation due to a simple misunderstanding among the authors. The trial was however registered before the results of the trial were in any way available to the authors and there were no changes made from the original protocol or the published study protocol. The trial is registered at clinicaltrials.gov with the identifier NCT02698319 (https://clinicaltrials.gov/ct2/show/NCT02698319).

## Results

**Fig 1:**
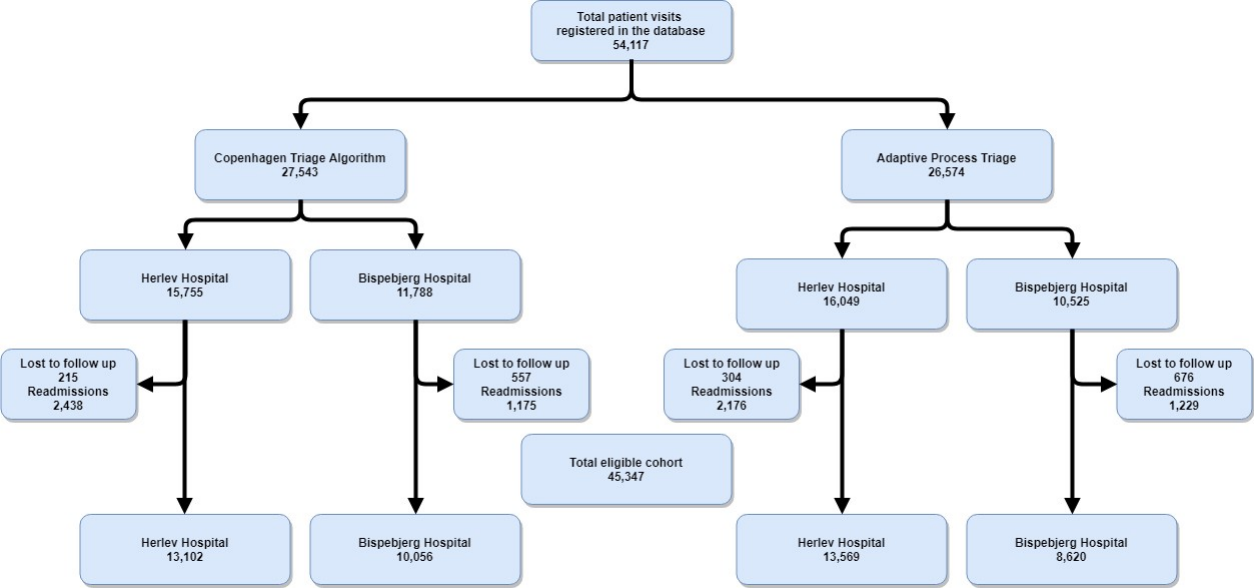
CONSORT diagram.

A total of 54,117 patient visits were included in the study between March 1^st^ 2015 and January 31^st^ 2016, and we concluded follow-up on May 1^st^ 2016. A total of 1,752 (3.2%) patient visits were excluded due to either erroneous or temporary identification in the ED, and a further 7,018 (13%) patient visits were excluded as readmissions within 90-days of the index admission, resulting in a final study population of 45,347 patient visits. Of these, 23,158 (51%) of these were triaged using CTA, 22,189 (49%) were triaged using ADAPT, and Herlev and Bispebjerg Hospitals had 26,671 and 18,676 patients included in the analysis, respectively (Fig 1). Table 1 and S1–S4 Tables summarize baseline characteristics and vital signs of the study population.

**Table 1:** Baseline characteristics divided into triage levels. Baseline characteristics and vital signs for patients admitted to the emergency department and triaged with two different systems., mean values (Standard deviation) across triage levels, arterial oxygen saturation: median (IQR).

	Copenhagen Triage Algorithm (CTA) n = 23,158	Adaptive Process Triage (ADAPT) n = 22,189	P
**Triage: Red (most urgent), n (%)**	623 (2.7)	695 (3.13)	0.0051
Female, n (%)	283 (45.4)	325 (46.8)	
Age, years,	62.6 (21.5)	62.1 (21.2)	
Systolic Blood Pressure, mmHg	134 (35)	136 (35)	
Heart rate, pr. min	99 (30)	106 (32)	
Respiratory rate, pr. min	23 (8)	24 (9)	
Arterial oxygen saturation, %	97 (93–99)	96 (92–98)	
Temperature, degrees Celsius	36.8 (1)	36.9 (1)	
**Triage: Orange, n (%)**	3,799 (16.4)	6,438 (29.0)	<0.001*
Female, n (%)	1,865 (49.1)	3,345 (52.0)	
Age, years	58.4 (21.7)	56.9 (22.1)	
Systolic Blood Pressure, mmHg	140 (30)	141 (28)	
Heart rate, pr. min	90 (23)	88 (22)	
Respiratory rate, pr. min	19 (5)	18 (4)	
Arterial oxygen saturation, %	97 (95–99)	97 (96–99)	
Temperature, degrees Celsius	36.7 (1)	36.8 (1)	
**Triage: Yellow, n (%)**	8,823 (38.1)	8,357 (37.7)	0.34
Female, n (%)	4,545 (51.5)	4,315 (51.6)	
Age, years	55.7 (22.0)	56.4 (22.1)	
Systolic Blood Pressure, mmHg	139 (26)	138 (25)	
Heart rate, pr. min	87 (19)	85 (18)	
Respiratory rate, pr. min	18 (3)	17 (3)	
Arterial oxygen saturation, %	97 (96–99)	97 (96–99)	
Temperature, degrees Celsius	36.7 (1)	36.9 (1)	
**Triage: Green (least urgent), n (%)**	9,403 (40.6)	6,677 (30.1)	<0.001*
Female, n (%)	4,782 (50.9)	3,473 (52.0)	
Age, years	56.4 (22.0)	56.7 (21.6)	
Systolic Blood Pressure, mmHg	139 (23)	138 (24)	
Heart rate, pr. min	82 (15)	82 (15)	
Respiratory rate, pr. min	17 (2)	17 (2)	
Arterial oxygen saturation, %	97 (96–99)	98 (96–99)	
Temperature, degrees Celsius	36.8 (1)	36. (1)	
**Missing triage**	**510 (2.2)**	**22 (0.10)**	<0.001*

**Fig 2:**
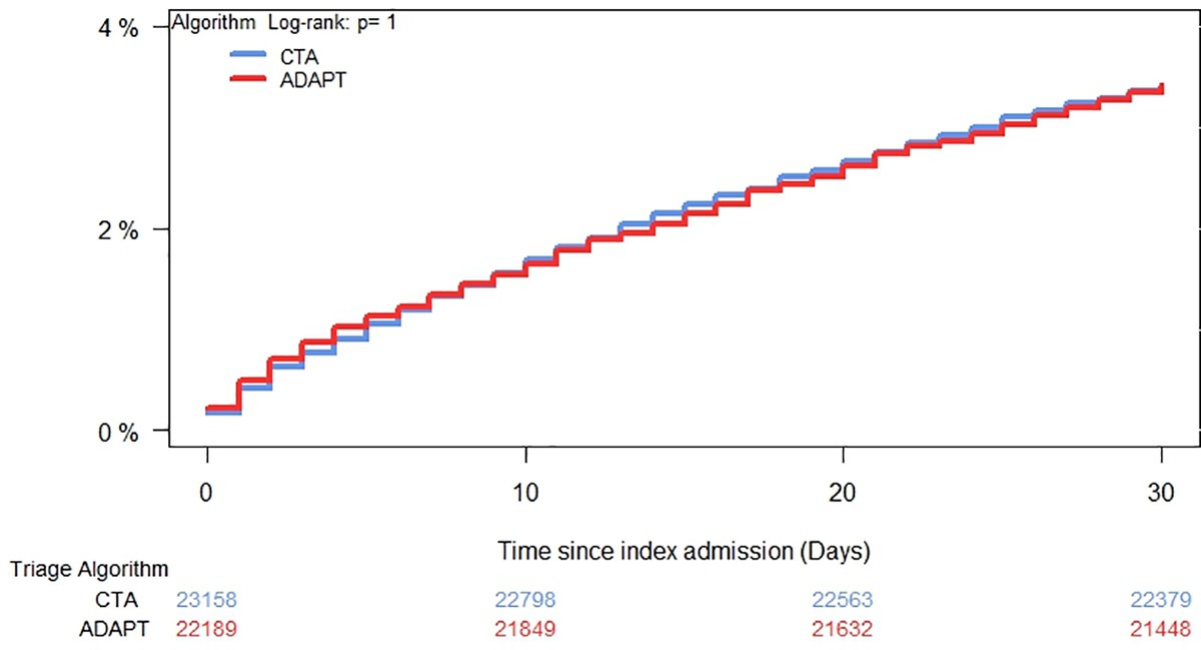
Cumulative incidence plot. 30-day all-cause mortality for patients admitted to the emergency department and triaged with two different systems.

At 30 days, death occurred in 793 (3.42%) patients in the CTA group and in 760 (3.43%) in the ADAPT group (P = 1.00), corresponding to a mortality difference of 0.01% (95% CI -0.336–0.334). The lower boundary of the 95% two-sided confidence interval for the difference between the occurrences of the primary endpoint in the CTA and ADAPT cohorts does not include–Δ (0.5%), therefore this met the pre-specified non-inferiority conditions. We observed no differences in the secondary endpoint of acute mortality (48 hours), in which mortality was 0.62% and 0.71% in patients triaged using CTA and ADAPT, respectively (P = 0.26), corresponding to a difference of -0.086 (95% CI -0.235 to 0.064). Similarly, we observed no differences for long-term mortality (90 days), 6.38% and 6.61%, respectively (p = 0.323), corresponding to a difference of -0.229 (95% CI -0.683 to 0.225). Cumulative incidence for 30-day mortality is plotted in Fig 2 and shows no difference between the two triage algorithms (P = 1.00).

**Fig 3:**
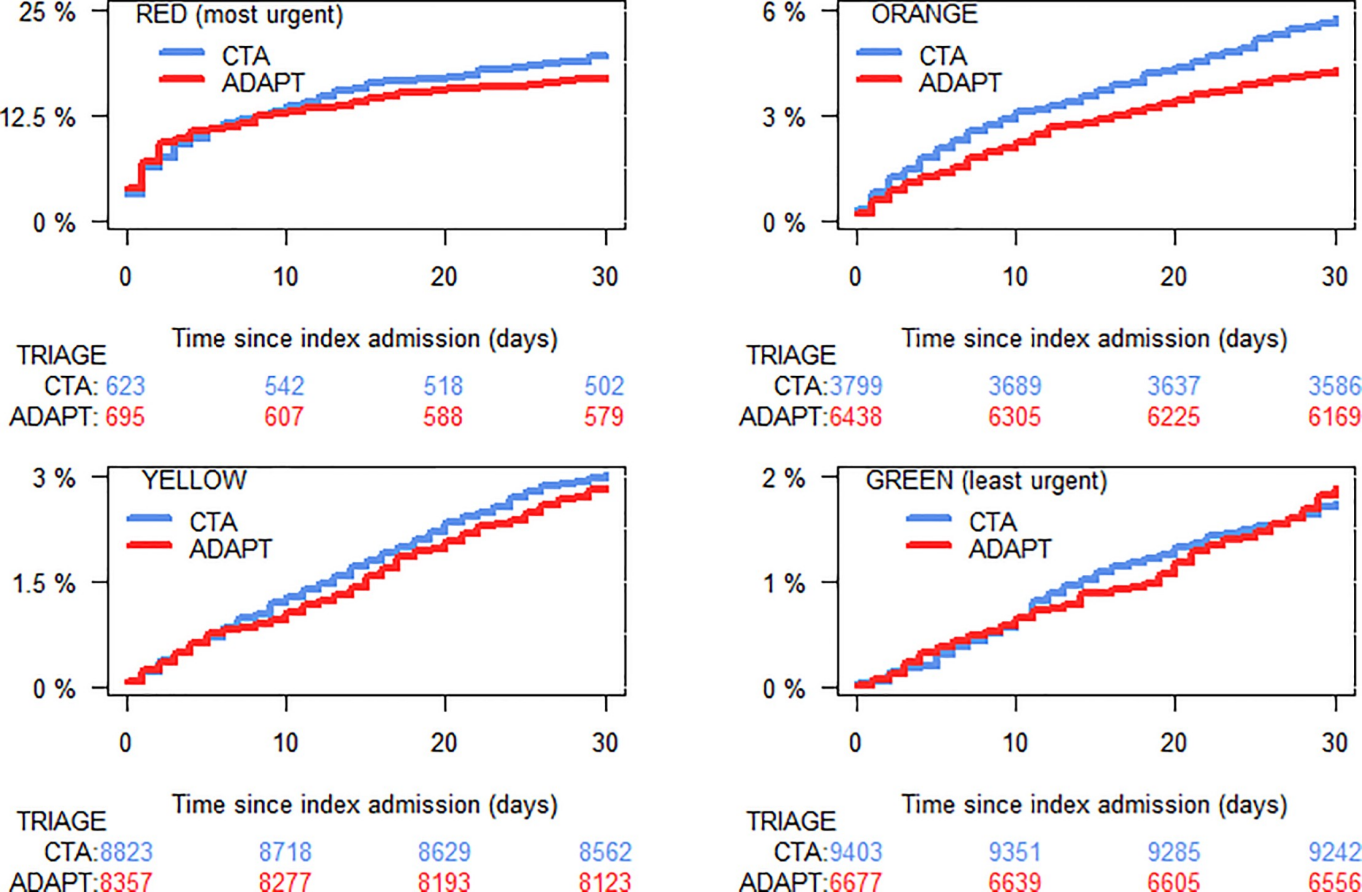
Cumulative incidence. 30-day all-cause mortality and distribution of triage levels for patients admitted to the emergency department.

The triage levels of the patients and corresponding cumulative 30-day mortality incidence for each triage level are summarized in Fig 3. Although the CTA group patients triaged at a significantly lower average urgency (P<0.001, Table 1), there was no significant increase in mortality observed among patients in the low-risk groups (3/yellow and 4/green). The moderate-to-high risk patients (2/orange) had a higher 30-day mortality in the CTA group (P<0.001).

The Receiver Operating Characteristics (ROC) curves indicate that CTA was non-inferior at 1, 2 and 7 days, and superior at 30, 60, and 90 days in predicting mortality relative to ADAPT (S2 Fig). The AUC for mortality at 30 days for CTA was 0.67 (95% CI 0.65–0.69) and 0.64 (95% CI 0.62–0.66) for ADAPT (P = 0.03). At 2 days AUC was 0.79 (95% CI 0.75–0.83) and 0.80 (95% CI 0.76–0.83) for CTA and ADAPT (P = 0.91), and at 90 days the AUC was 0.61 (95% CI 0.59–0.62) and: 0.57 (95% CI 0.56–0.59) for CTA and ADAPT, respectively (P = 0.002). Using the Youden index we found a cut-off of 2.5 yielding a specificity of 0.81 (95% CI 0.81–0.82), sensitivity of 0.44 (0.41–0.48), positive predictive value of 0.077 (0.071–0.083) and negative predictive value of 0.98 (0.98–0.98) for CTA. The corresponding values for ADAPT was a specificity of 0.69 (95% CI 0.68–0.69), sensitivity of 0.52 (0.49–0.52), positive predictive value of 0.055 (0.052–0.059) and negative predictive value of 0.98 (0.97–0.98). Further, the Brier scores of CTA and ADAPT were 0.032 and 0.033 respectively, both corresponding to acceptable performance.

**Fig 4:**
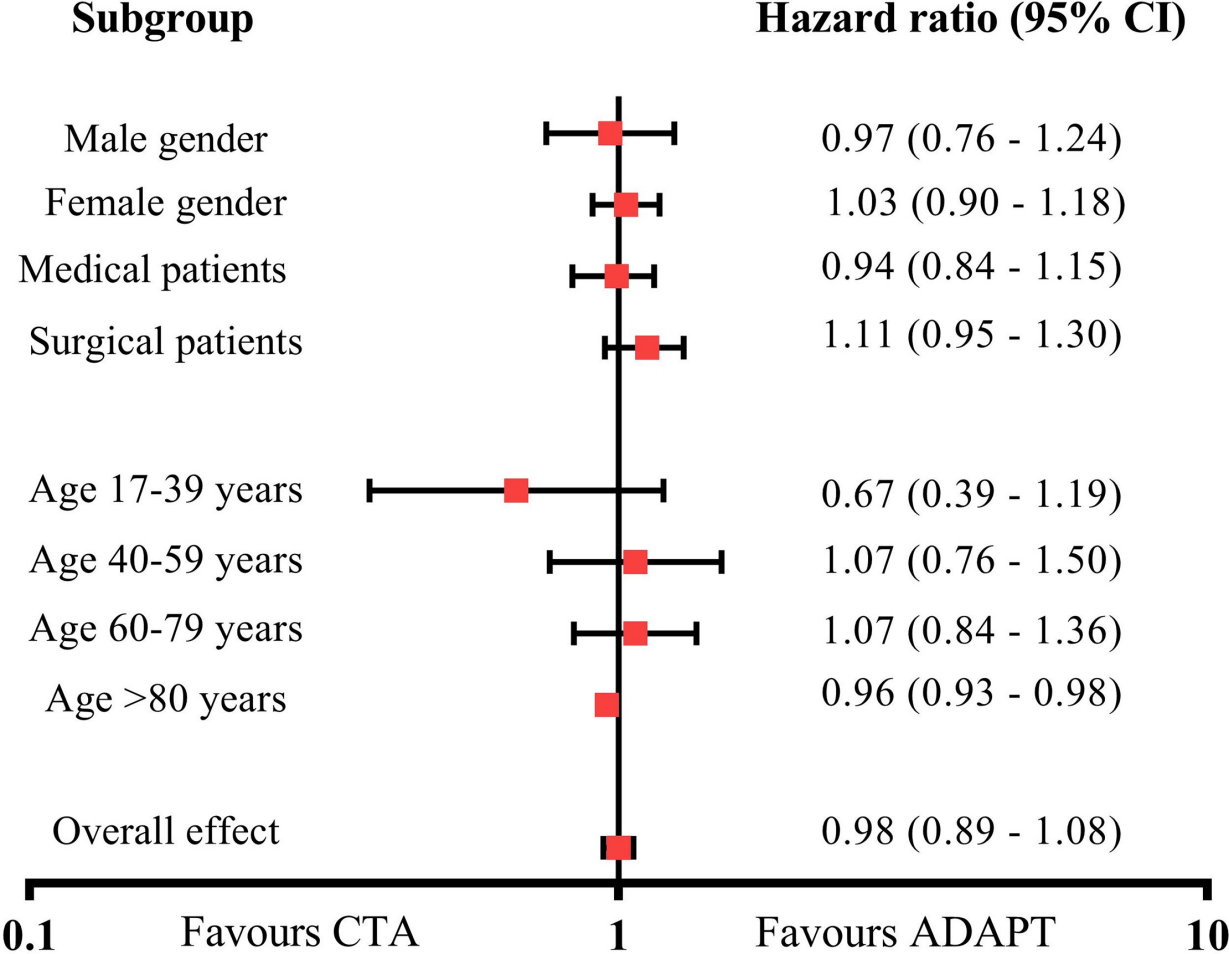
Forest plot for 30-day all-cause mortality for patients admitted to the emergency department.

A Forest plot revealed no significant differences between triage algorithms in risk categorized by sex, age group or medical/surgical patients (Fig 4). A sensitivity analysis that included vital signs, age, and sex showed consistent results and no added risk among patients in the CTA arm for mortality at 30 days (HR 1.05, 95% CI 0.92–1.20) or 90 days (HR 0.98, 95% CI 0.89–1.09) and showed a slight but significantly lower risk using CTA at 48 hours (HR 0.92, 95% CI 0.91–0.93). Notably, sensitivity analyses on the primary endpoint of 30-day mortality stratified by triage level revealed an increased risk in levels 1–3 in the triage comparison, indicating that CTA is superior in identifying patients at risk of short term death (S3 Fig).

Secondary end-points of various flow measures showed no significant differences between the two systems with regards to rate of admission to the intensive care unit at 0.86% and 0.82% (P = 0.59), average length of stay at 2.6 and 2.6 days (P = 0.32) and readmissions within 30 days at 7.61% and 7.23% (P = 0.10) or 90 days at 13.5% and 13.3% (P = 0.52) for CTA and ADAPT respectively. Similarly, there was no difference in waiting time from triage to treatment in either ED with waiting time at Bispebjerg Hospital at 67 min and 66 min (P = 0.50) and at Herlev Hospital at 40 min and 38 min (P = 0.50) for CTA and ADAPT, respectively.

We assessed an additional per-protocol cohort that excluded patients with no or erroneous triage (n = 532 (1.17%)). The 30-day mortality was 3.40% (n = 770) in patients triaged with CTA (n = 22,648), compared to 3.42% (n = 759) in patients triaged with ADAPT (n = 22,167), a difference of 0.024 (95% CI -0.360–0.312), HR 0.99 (95% CI 0.93–1.06).

In post hoc analyses we found no significant differences in in hospital mortality at 137 (0.60%) and 136 (0.64) (p = 0.58) or proportion of patients who left without being seen by a doctor 166 (0.73%) and 147 (0.70%) (P = 0.68) for CTA and ADAPT.

## Discussion

This is the first randomized study that evaluates different triage systems in relation to clinical endpoints. We find that the Copenhagen Triage Algorithm (CTA), a new and simpler triage algorithm with an emphasis on basic clinical assessment, is non-inferior to a systematic triage algorithm in determining patient outcomes. CTA produced a significantly lower level of triage on average and performed significantly better at predicting mortality risk in a large and unselected cohort of ED patients. There were no significant differences with regards to measures of patient flow.

These results indicate that the simpler CTA algorithm may safely replace more cumbersome systematic triage systems without a risk of harming patients. CTA could potentially lessen the resources used on triage.

A potential problem with any randomized trial of triage could be the positive impact of over-triage. It is possible that classifying more patients as Red or Orange (the most acutely ill patients) could lead to a lower mortality simple by more immediate treatment of those patients. However, this was not the case in this study, as CTA has significantly fewer patients triaged Orange while still being non-inferior to ADAPT on all-cause mortality. The reasons for this could be that fewer patients classified as acutely ill lead to lesser crowding in the ED.

We observed a significantly higher mortality rate among patients triaged as Orange using CTA than ADAPT. This is not because of differences in care, as the two systems have the same treatment algorithm (e.g. maximum waiting time to see a doctor). More likely it is because fewer low-risk patients were misclassified as Orange and instead correctly classified as Green or Yellow (less acutely ill). This can be seen by the significantly increased number of Green patients in the CTA cohort as compared to ADAPT, while the mortality rate of the Green patients remained the same.

We used a local adaption of the internationally used ADAPT system as the comparison triage system in this study. Like most other triage systems, the ADAPT system determines estimated urgency using a list of vital signs (e.g. heart rate) and a series of flow-charts describing chief complaints (e.g. dyspnea) [6], and assigns a color that determines the patient’s urgency in seeing a physician. ADAPT is largely similar to most other widely used triage models, including the Manchester Triage System, The Canadian Triage and Acuity Scale, and the Australasian Triage Scale [3, 4, 15, 16], both in terms of composition and distribution of patients at each triage level [17, 18] as well as mortality rates within these categories [6]. The follow-up intervals, procedures and overall terminology was the same in both CTA and ADAPT to ensure that the two systems were directly comparable.

Specific symptoms are well-known predictors of disease and prognosis [17, 19] but attempts to quantify and list these in triage often lead to challenges, including trouble grading the severity of the symptoms as well as difficulty characterizing every possible symptom or symptom combination, and there is little evidence or agreement across triage systems [20]. Lending more weight to a basic clinical assessment could potentially eliminate these problems as seen in the CTA, in which the ED nurse had the ability to reclassify patients as more urgent (up to 2 levels) or less urgent (1 level) with no explanation required. Most triage models, including ADAPT, permit ED nurses to reclassify patients as more urgent, but few incorporate the clinical assessment as an integral part of the algorithm despite recent data supporting the use of clinical assessment even by relatively untrained staff [21, 22]. An exception is the Emergency Severity Index where the ED nurse separates the acutely ill (level 1 and level 2) from the remaining patients (levels 3–5) which are then sorted based on resource utilization [23].

The determination of the outcome measures that most accurately evaluate a triage system remains an open question. The ideal gold standard for comparison is a well-validated model, but all current triage systems were implemented prior to validation [5, 20]. A useful clinical outcome should be independent from the results of triage, and the patient’s triage category could affect hospitalization or admittance to the intensive care unit (ICU) [24] (e.g. the patient was triaged as acutely ill and was therefore admitted to the ICU), making those outcomes less useful. Mortality rate was chosen as the primary end-point of this study for this reason. We also report other more patient flow related outcome measures, e.g. admittance to the ICU, patient length of stay as well as the number of patients that left the ED without being seen by a doctor. Other factors that could be considered for an evidence-based triage model include the effect on crowding [2] and model reliability as determined by inter- and intra-rater agreement [15, 20] as well as the triage system’ ability to predict other markers of acute illness such as team activation and radiological examinations or specific procedures and treatments (e.g. thrombolysis).

### Strengths and limitations

The large and unselected nature of the cohort, including both medical and surgical patients of all ages is a strength of this study. However, individual randomization is not practical with an intervention like triage for several reasons. First, continuously switching between triage models could slow down the flow of the ED, which would cause crowding and putting the patients at risk. Second, such a setup runs the risk of bias, since nurses may default to one triage model over the other during busy times.

We chose cluster-randomization to avoid this. Cluster-randomized design introduces dependence between individual units sampled, which is largely alleviated by the cross-over in the study, and relies on a similar patient inclusion during both parts of the study. We ensured this by determining the total study period by the number of included patients rather than by time. We further note that the sensitivity and subgroup analyses performed all showed similar results.

## Conclusion

The Copenhagen Triage Algorithm, based on vital signs and a basic clinical assessment by an ED nurse, was non-inferior to a traditional triage algorithm in terms of short term mortality, and was superior at predicting mortality.

## Supporting information

S1 Checklist CONSORT extension for Cluster Trials Checklist (docx). (DOCX)

S1 Fig Diagram of triage for patients admitted to the emergency department using the CTA and ADAPT systems. (TIF)

S2 Fig Receiver operating characteristics. (TIF)

S3 Fig Forest Plot,the risk of 30-day all-cause mortality for patients admitted to the emergency department–Each triage level of CTA compared to each corresponding level of triage using ADAPT. (TIFF)

S1 Table Baseline characteristics divided into hospitals. Baseline characteristics for patients admitted to the emergency department, mean values (Standard deviation), arterial oxygen saturation: median (IQR).
(XLSX)

S2 Table Baseline chracteristics divided into triage systems. Baseline characteristics for patients admitted to the emergency department, mean values (Standard deviation), arterial oxygen saturation: median (IQR).
(XLSX)

S3 Table Baseline characteristics for Herlev Hospital divided among triage systems. Baseline characteristics for patients admitted to the emergency department, mean values (Standard deviation), arterial oxygen saturation: median (IQR).
(XLSX)

S4 Table Baseline characteristics for Bispebjerg Hospital. Baseline characteristics for patients admitted to the emergency department, mean values (Standard deviation), arterial oxygen saturation: median (IQR).
(XLSX)

S1 Protocol Published study protocol. (PDF)

S2 Protocol Study protocol. (DOCX)
