# Supplementary figures and images for: The Copenhagen Triage Algorithm is non-inferior to a traditional triage algorithm: A cluster-randomized study
Source: PLoS One. 2019 Feb 4;14(2):e0211769. doi: 10.1371/journal.pone.0211769 (PMC6361446; doi:10.1371/journal.pone.0211769)

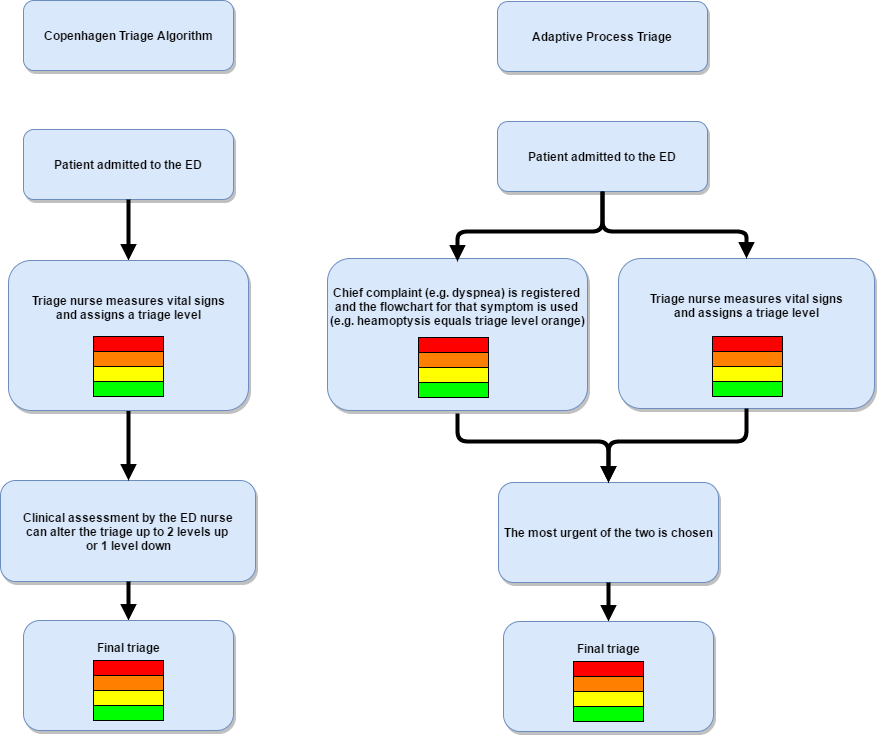

Supplement: S1 Fig — (TIF) [file pone.0211769.s002.tif]

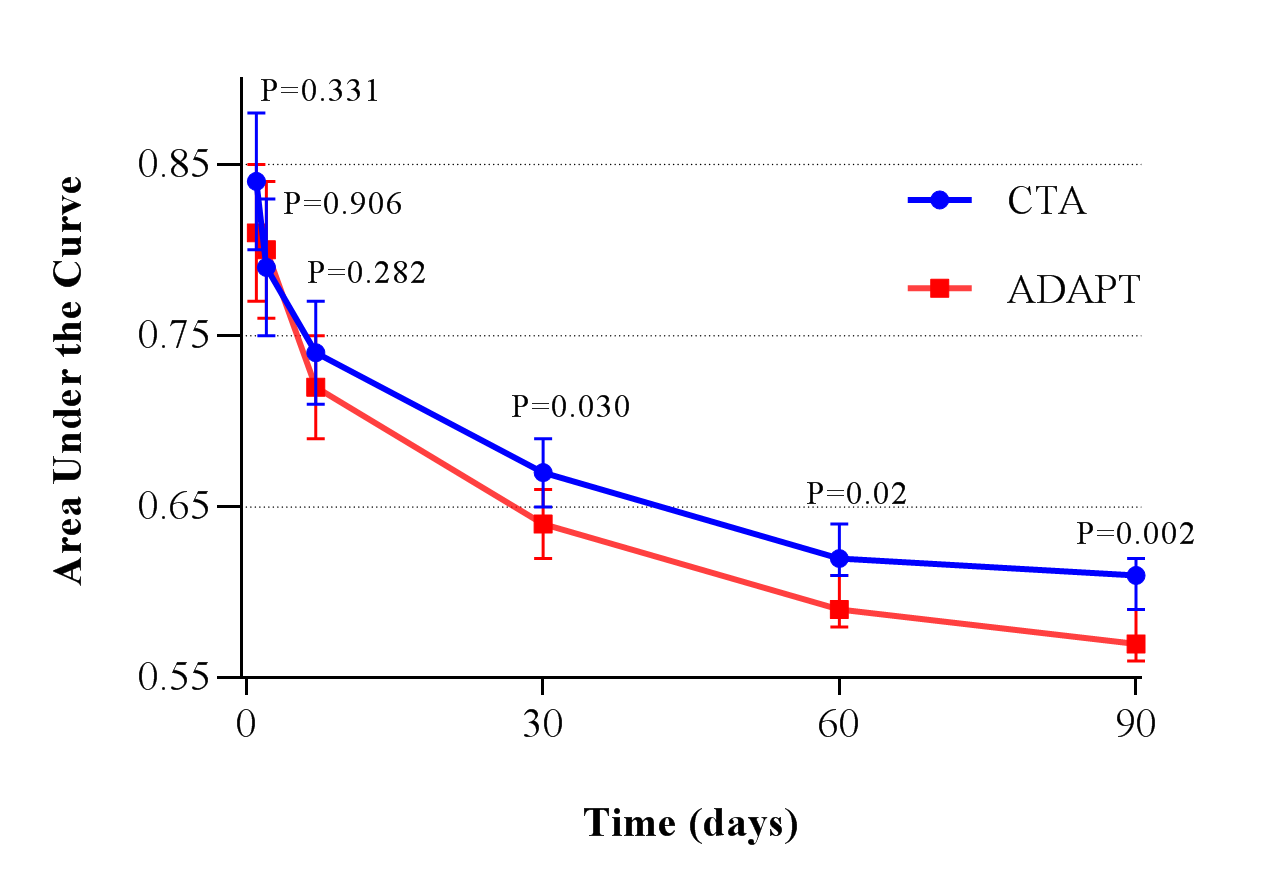

Supplement: S2 Fig — (TIF) [file pone.0211769.s003.tif]

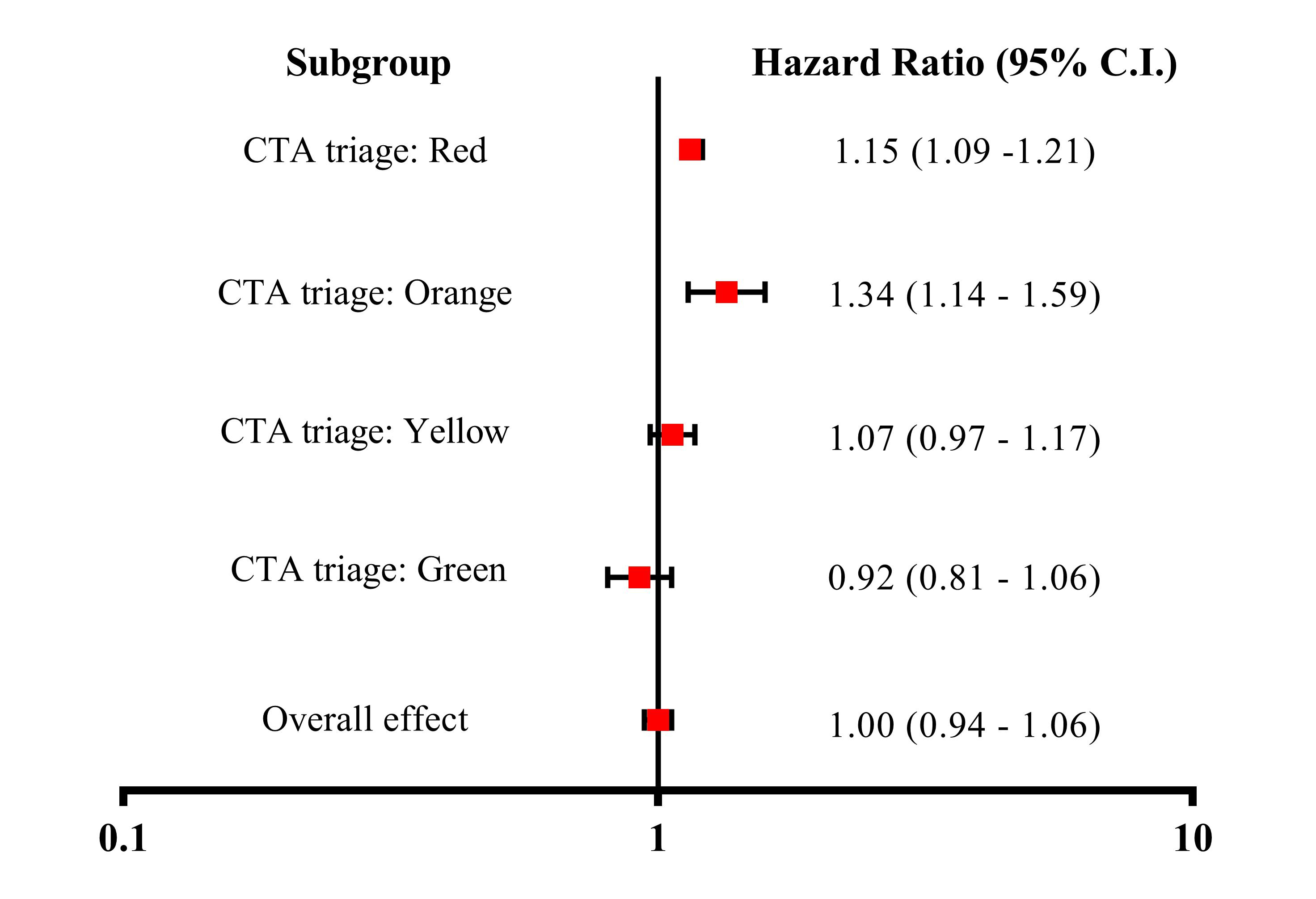

Supplement: S3 Fig — (TIFF) [file pone.0211769.s004.tiff]
