# Supplementary material for: The Copenhagen Triage Algorithm is non-inferior to a traditional triage algorithm: A cluster-randomized study
Source: PLoS One. 2019 Feb 4;14(2):e0211769. doi: 10.1371/journal.pone.0211769 (PMC6361446; doi:10.1371/journal.pone.0211769)
Supplement: S1 Protocol — (PDF) [file pone.0211769.s009.pdf]

Hasselbalch et al. Scandinavian Journal of Trauma, Resuscitation and Emergency Medicine (2016) 24:123
DOI 10.1186/s13049-016-0312-6

Scandinavian Journal of Trauma, Resuscitation and Emergency Medicine

STUDY PROTOCOL Open Access

The Copenhagen Triage Algorithm: a randomized controlled trial

Rasmus Bo Hasselbalch[1*], Louis Lind Plesner[1], Mia Pries-Heje[2], Lisbet Ravn[2], Morten Lind[2], Rasmus Greibe[3], Birgitte Nybo Jensen[4], Lars S. Rasmussen[5] and Kasper Iversen[1,2]

Abstract

Background: Crowding in the emergency department (ED) is a well-known problem resulting in an increased risk of adverse outcomes. Effective triage might counteract this problem by identifying the sickest patients and ensuring early treatment. In the last two decades, systematic triage has become the standard in ED's worldwide. However, triage models are also time consuming, supported by limited evidence and could potentially be of more harm than benefit. The aim of this study is to develop a quicker triage model using data from a large cohort of unselected ED patients and evaluate if this new model is non-inferior to an existing triage model in a prospective randomized trial.

Methods: The Copenhagen Triage Algorithm (CTA) study is a prospective two-center, cluster-randomized, cross-over, non-inferiority trial comparing CTA to the Danish Emergency Process Triage (DEPT). We include patients ≥16 years (n = 50.000) admitted to the ED in two large acute hospitals. Centers are randomly assigned to perform either CTA or DEPT triage first and then use the other triage model in the last time period. The CTA stratifies patients into 5 acuity levels in two steps. First, a scoring chart based on vital values is used to classify patients in an immediate category. Second, a clinical assessment by the ED nurse can alter the result suggested by the score up to two categories up or one down. The primary end-point is 30-day mortality and secondary end-points are length of stay, time to treatment, admission to intensive care unit, and readmission within 30 days.

Discussion: If proven non-inferior to standard DEPT triage, CTA will be a faster and simpler triage model that is still able to detect the critically ill. Simplifying triage will lessen the burden for the ED staff and possibly allow faster treatment.

Trial registration: Clinicaltrials.gov: NCT02698319, registered 24. of February 2016, retrospectively registered

Keywords: Triage, Emergency Department, Vital signs

Background

Crowding in emergency departments (ED) poses a serious patient safety concern in hospitals worldwide [1]. Since not every patient can be seen by a doctor on arrival, ED's prioritize patients according to their perceived urgency of care. In most hospitals today, this prioritization is done by systematic triage [2]. Patients are classified on the basis of a pre-specified algorithm into 5 acuity groups according to vital signs and primary symptom. Before systematic triage was introduced, a simple clinical assessment was used to prioritize the succession in which patients were seen by doctors [2].

The first systematic triage model was introduced in a local hospital in Australia in the 1970s, and since then 4 triage models have become widespread: Australasian Triage Scale (ATS), Canadian Triage and Acuity Scale (CTAS), Manchester Triage System (MTS), and Emergency Severity Index (ESI) [2–5]. An overview of existing models is provided in Table 1. These models are widely used in their countries of origin, but they have also been implemented to varying degrees across the world, where local adaptations has been done [6–9]. In Scandinavia, Sweden was the first to focus on triage developing two different models: The Medical Emergency Triage and Treatment System and Adaptive Process Triage (ADAPT) [10].

* Correspondence: r.hasselbalch@gmail.com
[1]Department of Cardiology, Herlev-Gentofte Hospital, Copenhagen, Denmark
Full list of author information is available at the end of the article

BioMed Central

© 2016 The Author(s). **Open Access** This article is distributed under the terms of the Creative Commons Attribution 4.0 International License (http://creativecommons.org/licenses/by/4.0/), which permits unrestricted use, distribution, and reproduction in any medium, provided you give appropriate credit to the original author(s) and the source, provide a link to the Creative Commons license, and indicate if changes were made. The Creative Commons Public Domain Dedication waiver (http://creativecommons.org/publicdomain/zero/1.0/) applies to the data made available in this article, unless otherwise stated.

Table 1 An overview of existing triage models

	DEPT	ATS	MTS	ESI	CTAS
Introduction	2009	1994	1996	1999	1995
Levels of triage	5	5	5	5	5
Time to contact with doctor	0 - 15 - 60 - 180 - 240 min	0 - 10 - 30 - 60 - 120 min	0 - 10 - 60 - 120 - 240 min	0 min - NS	0 - 15 - 30 - 60 - 120 min
Vital signs	SAT, HR, BP, GCS, TP, RR	BP, GCS, RR, HR, SAT, BSL	Varies	HR, SAT, RR - TP[a]	Varies
List of primary complaint	Yes	Yes	Yes	No	Yes
Other factors				Resources needed	
Inter-observer variability (Kappa/percentage)		38,7–79 %	*K* 0,48	*K* 0,78[b]	*K* 0,2-0,84
Mortality[c]	11 - 2,2 - 1,2 - 0,5 % - NS	12 - 2 - 1 - 0,3 - 0,03 %	10 - 0,04 - 0004 - 0002 - 0 %	25 - 4 - 2 - 1 - 0 %	22 - 0,22 - 0031 - 0018 - 0 %
LOS				4,7 - 4,3 - 4,3 - 7,3 - 2,0 days	

DEPT Danish Emergency Process Triage, *ATS* Australasian Triage Scale, *MTS* Manchester Triage Scale, *ESI* Emergency Severity Index, *CTAS* Canadian Triage and Acuity Scale, *SAT* Blood Oxygen Saturation, *HR* Heart Rate, *BP* Blood Pressure, *GCS* Glascow Coma Scale, *TP* Temperature, *RR* Respiratory Rate, *BSL* Blood Sugar Level, *NS* Not specified, *LOS* length of stay
[a]Only measured for patients under < 3 years of age
[b]Weighted Kappa
[c]Mortality measures were for different time intervals DEPT - In hospital, ATS - 24-h, MTS - In ED, ESI - 60 day, CTAS - In ED

ADAPT is rather similar to CTAS and ATS and it focuses on vital signs and the patient's presenting complaint.

In Denmark triage has been broadly implemented over the last decade [11]. Most ED's use a slightly modified version of ADAPT called Danish Emergency Process Triage (DEPT) [11–14].

Most triage models have been developed on the basis of expert opinion and they are not based on data from large prospective cohorts [15]. The models have then been implemented and retrospectively validated [5, 16–18]. The validation has focused on the respective models' inter-observer variability [15, 19] and ability to predict length of stay [20] and mortality [19, 21–23] but there are no prospective studies addressing if the models can prevent serious adverse events. Systematic triage takes time and prospective studies are needed to determine the future of ED triage [18].

Recently, data from ED visits have been collected over a period of 3 months at North Zealand Hospital giving a complete cohort of 6 000 patient ED visits [24]. This study confirmed earlier studies' conclusion that vital signs at admission are strong predictors of acute patient outcomes [21], and further demonstrated that a quick clinical assessment of patients by phlebotomists and medical students was superior in predicting 30-day mortality than formalized DEPT triage [25]. Based on these findings, our hypothesis was that a simple model of vital signs could be constructed that would perform as well as DEPT when combined with a quick clinical assessment by the ED nurse.

The aim of this article is to describe the design of the CTA, how the algorithm was designed and how it will be tested in a randomized controlled trial.

Design

The study is a prospective two-center, cluster-randomized, cross-over, non-inferiority trial. CTA is compared to the preexisting DEPT. The primary aim of the CTA is to assess if it improves patient outcome, secondarily to obtain a faster ED acuity stratification. The primary end-point is 30-day mortality and secondary end-points are length of stay (LOS), time to treatment, admission to intensive care unit (ICU), and readmission within 30 days. Follow-up information will be attained from central registries.

Setting and interventions

Two equally sized ED's in the capital region of Copenhagen are recruited to perform the study; Herlev Hospital and Bispebjerg Hospital with 70.000 and 85.000 annual admissions, respectively [26, 27]. Both centers are 24-h acute care hospitals offering broad medical, surgical, neurological, level-2 trauma and ICU services. The centers are randomly assigned to perform either CTA or DEPT triage as primary evaluation of patients. Subsequently, both hospitals will cross-over to use the other model. The number of admissions with CTA and DEPT will be 1:1. The CTA will be implemented during 2 weeks before initiation of data collection.

Patient participation

In the study period, all patients admitted at any of the two centers will be included. Admission was defined as referral to a bed in the ED. The study design allows inclusion rate and adherence to the intervention to be close to 100 % as the two triage models will not be used at the same time and all acutely admitted patients are

categorized using triage. However, patients ≤ 16 years gynecology and obstetric patients will not be included since they are admitted directly to the respective department. Patients where major trauma is suspected prehospital will be admitted to a tertiary center in the region.

Copenhagen Triage Algorithm

The CTA is based on vital signs and a clinical assessment by ED nurses.

The CTA classifies patients as red (resuscitation), orange (emergent), yellow (urgent), green (non-urgent) or blue (minor injuries and complaint) and this is done in two steps. A scoring chart based on vital signs is used to classify patients in an immediate category but a clinical assessment can alter the assigned category suggested by the score up to two classed up or one class down. The model is illustrated in Fig. 1. DEPT has a similar structure, but the classification depends on a more complex algorithm designed to classify patients based on their primary complaint (symptom) as well as vital signs [14]. DEPT only allows the ED nurses to reclassify patients as more urgent, not less. There will be no reclassification for the blue triage category as it is defined in in the same way using CTA and DEPT for patients with minor injuries or complaints.

The cut-off points for vital signs (Fig. 1) of CTA were calculated using the results of the TRIAGE database [24]. This was done by defining either the 10 % highest (e.g. heart rate) or the 10 % lowest (e.g. systolic blood pressure) as abnormal, and using univariate logistic regression to determine its ability to predict 30-day mortality. The significant factors were then combined in a multivariate logistic regression with backwards elimination to determine the independent variables and their odds ratio in predicting 30-day mortality. These odds ratios were rounded to their nearest integer and combined to form a score.

The significant factors were systolic blood pressure < 100 mmHg, heart rate > 110 bpm, respiratory rate > 22 min^{-1}, arterial oxygen saturation < 94 %, and treatment with oxygen (Fig. 1). Final categories were defined so that green patients had a 30-day mortality below 2.5 %, yellow patients had a 30-day mortality between 2.5 and 7.5 %, orange patients had a 30-day mortality between 7.5 and 15 % and red patients had a 30-day mortality above 15 %.

Results of the primary regression analyses using these cutoffs are shown in Table 2. Receiver-operating characteristics (ROC) curves showed that CTA and a quick clinical assessment performed by phlebotomists and medical students were superior to DEPT in identifying death within 30 days (Fig. 2). Area under the curve (AUC) statistics for the two models are presented in Table 3, which show that the CTA score is significantly superior to DEPT in predicting the risk of 30-day mortality.

Sample size

The trial is designed as a non-inferiority trial, i.e to show that 30-day mortality using CTA is not higher than using DEPT. The 30-day mortality in all admitted patients in the TRIAGE database was 4.2 % [24] and the 28-day mortality in the Acute Admission Database was 4.2 % [28]. Hence, we estimated the 30-day mortality to be 4.2 % for this study. Due to the estimated low incidence of the primary end-point, the proportional difference between the CTA and DEPT group (non-inferiority margin (Δ)) is set at 0.5 %. The power to confirm non-inferiority is set at 80 % with a two-sided confidence interval of 95 %, which would require a sample size of 39.820. We chose to include a minimum of 40.000 patients. The combined percentage of admission to intensive or semi-intensive care unit in the TRIAGE database was 3.4 % [24], i.e sample size should be adequate to prove non-inferiority for this secondary end-point as well. Reported 30-day readmission rate is usually around 10 % [29] meaning sample size will likely be sufficient to prove non-inferiority. In calculating the primary end-point of 30-day mortality, patients readmitted more than 30 days after discharge will be considered as separate encounters.

Statistics

Non-inferiority will be considered to have been confirmed if the lower bound of the 95 % two-sided confidence interval for the difference between the occurrence

Factor		Points	Red	Orange	Yellow	Green
Oxygen treatment	+	4	8-14 points	4-7 points	2-3 points	0 points
Systolic blood pressure	< 100 mmHg	3	Rescuscitation	Emergent	Urgent	Non-urgent
Respiratory rate	> 22 min^{-1}	3	Life threatened	Critical illness	Potentially unstable	Stable
Heart rate	> 110 min^{-1}	2	Constant	Re-evaluation	Re-evaluation	Re-evaluation
SpO_2	< 94 %	2	Re-evaluation	Every 15 min	Every 60 min	Every 180 min
A clinical assessment on arrival: Triage nurse can move the patient one triage level down or up to two levels up based on his/her own evaluation of symptoms			Red	Orange	Yellow	Green

Fig. 1 The Copenhagen Triage Algorithm

Table 2 Results of the multiple logistic regression using the CTA cut-off values on the TRIAGE database

	Odds ratio	*P*
Oxygen treatment	4,27	<0,01
Systolic BP < 100	3,28	<0,01
Heart rate > 110	2,14	<0,01
Respiratory rate > 22	2,71	<0,01
SpO2 < 94 %	1,67	<0,01

of the primary endpoint in the CTA and DEPT cohorts does not include -Δ. Furthermore, CTA could show superiority over DEPT; this will be considered when the lower bound of the confidence interval mentioned above exceeds 0. This analysis will only be performed if non-inferiority is confirmed. The power to demonstrate that CTA is superior will be 0.72 with a sample size of 40.000, while there will be a 5 % chance that DEPT triage is better. LOS and time-to-treatment in the two models will be log-transformed and compared using parametric tests. The patient populations included with CTA and DEPT will be compared using standard parametric tests.

Timeline

After a 1 week learning period for the staff, the study was initiated the 1st of March, 2015. Herlev Hospital was selected to begin with CTA, i.e. Bispebjerg Hospital continued their use of DEPT triage. This continued until the 17th of August 2015. Subsequently, the hospitals crossed-over. By the end of January 2016 the inclusion

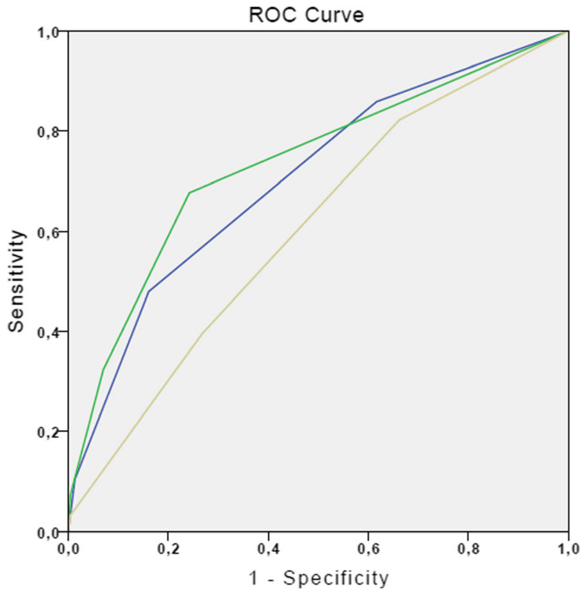

Fig. 2 Receiver-Operating Characteristics for DEPT, CTA and a quick clinical assessment in the TRIAGE database. Blue—Clinical assessment; Yellow—DEPT; Green—CTA

Table 3 Area under the curve results for the Receiver-Operating Characteristics for DEPT, CTA and a quick clinical assessment in the TRIAGE database

	AUC	Standard error
CTA	0735	0,02
Clinical assessment	0705	0,02
DEPT	0605	0,02

stopped as planned. A total of approximately 50,000 patient visits have been recorded.

Discussion

Emergency department (ED) triage impacts most patients and it can be a valuable tool for managing a crowded and busy ED. However, existing models of triage are supported by limited evidence. The Copenhagen Triage Algorithm (CTA) aims to be a faster and better way to identify acutely ill patients as well as the less urgent patients in the ED. The CTA Study is a randomized trial comparing CTA to the standard Danish Emergency Process Triage (DEPT) in an unselected population. To the best of our knowledge, this is the first study to prospectively evaluate a triage model in a randomized study. If the hypothesis of non-inferiority in detecting mortality is confirmed CTA will provide less resource consuming method of triage in the ED while still identifying patients in need of urgent care.

The time and resources used on systematic triage is not without a price as crowding in the ED is a well-established risk that can compromise patient safety [1]. Overcrowding in the ED happens when the patient load exceeds the available recourses. This situation leads to increased length of stay and mortality across patients both high and low risk [30, 31]. A retrospective study of visits to the Canberra Hospital ED [31] showed that patients presenting to the ED during “overcrowded shifts” had a 34 % higher risk of 10-day in hospital mortality than patients presenting during normal shits. This risk persisted even when accounting for level of triage (by Australasian Triage Scale) suggesting a level of under-triage during crowded shifts [31]. A systematic review of crowding in the ED [30] found ED bottlenecks to be common in the literature often arising from inadequate staffing. As triage by definition is time and resource consuming, speeding up this process alone could help alleviate some of these problems.

Compared to other triage models CTA is a vastly simpler model comprised of vital signs (blood pressure, heart rate, arterial oxygen saturation, respiratory rate, and the need for oxygen treatment) combined with a clinical assessment by the ED nurse. Since vital signs are measured as part of any patient admission except for minor injuries, the only additional time consuming part

of CTA is the clinical assessment which can be done in few minutes even by clinically uneducated staff [25]. This is contrasted by most existing triage models such as ATS, MTS, DEPT and CTAS that use extensive lists of primary complaint or causes of contact [14, 21] aimed at identifying potentially urgent patients. These aim to find the patients primary symptom and triage based on prior consensus of the urgency of symptoms. These are mostly based on expert opinion and has been shown to lead to over-triage, increasing the triage level for about half the patients while lowering the sensitivity and specificity of the final triage [21].

Abnormal vital signs are strong predictors of mortality in patients admitted through ED's [21]. Though smaller studies have shown that vital signs alone are insufficient to detect the critically ill [32, 33], combining these with a clinical assessment by the ED nurse may significantly improve this without using extra resources [25]. Which vital signs and cut-off points should be used is still a subject of debate. In most models, like the ATS, it is established by expert opinion [34] and a systematic review of the literature from 2011 by Farrohknia et al. [15] showed limited or insufficient evidence for any of the vital signs usually used in triage models (respiratory rate, arterial oxygen saturation, heart rate, and level of consciousness). Thus we decided to define new cut-off values based on the large cohort of patients we had gathered.

As the vital signs were meant to reflect acute illness, we chose not to include age as a factor in the score. Biomarkers were not a part of this study though several biomarkers have been shown to be strong predictors of mortality [35, 36]. In the future, a combined score of vital signs and biomarkers may be the most sensitive method of detecting the vast amount of non-urgent patients visiting the hospital every day [24, 37].

Limitations

The primary end-point is 30-day mortality which is associated with multiple factors besides the triage model used at admission. Mortality has been used previously to assess the efficacy of triage models [19, 21–23]. If CTA has any impact on acute mortality (e.g. 8 h) it would also affect 30-day mortality given it is the only intervention performed. However, if 8-h mortality was chosen as primary end-point, it would favor a model ranking a majority of patients in acute category and not necessarily reflect the impact on less acute patients who might die during hospitalization or within the first month. Admission to ICU as secondary endpoint was chosen to reflect a more immediate impact of triage.

In the acute care setting, it is not feasible to randomize individual patients for different triage models for several reasons. The individual randomization would be a huge logistical challenge in a busy ED and it would be associated with delays and crowding, which would be unethical. Furthermore, it is likely that the staff would prefer the faster algorithm (CTA) and tend to use that in most patients once it is available in the department. To avoid this, cluster-randomization was chosen. Cluster-randomized design introduces dependence between individual units sampled (e.g. difference in patient population, skill of ED nurses/physicians). This is largely alleviated by cross-over in the study and relies on a similar patient inclusion during both parts of the study. This is ensured by determining the total study period through number of included patients instead of time. Furthermore, the number of clusters were very small but the cross-over design partly compensates for this.

Readmissions after 30 days are also included which means patients can be exposed to both DEPT and CTA triage during the study period. This means patients with diagnoses prone to readmission will be overrepresented in the study. Moreover, if the inclusion rate differs significantly between the two hospitals during the study period this might slightly favor the model used in most patients late in the study due to lead-time bias. It is possible for patients to be admitted in both hospitals during the study period though not likely. Patients are not systematically transferred between these two hospitals so systematic bias is unlikely in this regard. A final limitation of the study is that patients with suspected major trauma are admitted to a tertiary center in the region, meaning that few such patients will be included. However, the major aim of the CTA study is not to focus on seriously injured patients as they are managed according to specific algorithms anyway.

Abbreviations

ADAPT: Adaptive Process Triage; ATS: Australasian Triage Scale; AUC: Area Under the Curve; BP: Blood Pressure; BSL: Blood Sugar Level; CTA: Copenhagen Triage Algorithm; CTAS: Canadian Triage and Acuity Scale; DEPT: Danish Emergency Process Triage; ED: Emergency Department; ESI: Emergency Severity Index; GCS: Glascow Coma Scale; HR: Heart Rate; LOS: length of stay; MTS: Manchester Triage Scale; NS: Not specified; ROC: Receiver-Operating Characteristics; RR: Respiratory Rate; SAT: Blood Oxygen Saturation; TP: Temperature

Acknowledgements

Not applicable.

Funding

The study is funded by TrygFonden. The trial is driven and initiated by the researchers and the foundation has no influence on study design, data analysis, interpretation or publication.

Availability of data and supporting material

When the study is completed, it will be located on a central server at Statistics Denmark in anonymized form. After analysis and publication of all outcomes the database will be available from the authors on reasonable request.

Authors' contributions

RBH, LP and KI drafted the manuscript. KI had the original idea for the study. All authors read and approved the final manuscript.

Competing interests
The authors declare that they have no competing interests.

Consent for publication
Not applicable.

Ethics approval and consent to participate
The study protocol was approved by the local ethics committees (H-4-2014-FSP). The Danish Data Protection Agency (HEH-2014-118) and the Danish Health and Medicines Authority (3-3013-1119/2). The study is registered on clinicaltrials.gov (NCT02698319).

Author details
[1]Department of Cardiology, Herlev-Gentofte Hospital, Copenhagen, Denmark. [2]Department of Emergency Medicine, Herlev-Gentofte Hospital, Copenhagen, Denmark. [3]Department of Cardiology, Bispebjerg Hospital, Copenhagen, Denmark. [4]Department of Emergency Medicine, Bispebjerg Hospital, Copenhagen, Denmark. [5]Department of Anaesthesia, Center of Head and Orthopaedics, Rigshospitalet, University of Copenhagen, Copenhagen, Denmark.

Received: 4 August 2016 Accepted: 30 September 2016
Published online: 10 October 2016

References

1. Carter EJ, Pouch SM, Larson EL. The relationship between emergency department crowding and patient outcomes: a systematic review. J Nurs Sch. 2014;46(2):106–15.
2. FitzGerald G, Jelinek GA, Scott D, Gerdtz MF. Emergency department triage revisited. Emerg Med J. 2010;27(2):86–92.
3. Weyrich P, Christ M, Celebi N, Riessen R. Triage systems in the emergency department. Med Klin Intensivmed Notfallmed. 2012;107(1): 67–78. quiz 9.
4. Wuerz RC, Milne LW, Eitel DR, Travers D, Gilboy N. Reliability and validity of a new five-level triage instrument. Acad Emerg Med. 2000;7(3):236–42.
5. Moll HA. Challenges in the validation of triage systems at emergency departments. J Clin Epidemiol. 2010;63(4):384–8.
6. Graff I, Goldschmidt B, Glien P, Bogdanow M, Fimmers R, Hoeft A, et al. The German Version of the Manchester Triage System and its quality criteria–first assessment of validity and reliability. PLoS One. 2014;9(2):e88995.
7. Bruijns SR, Wallis LA, Burch VC. A prospective evaluation of the Cape triage score in the emergency department of an urban public hospital in South Africa. Emerg Med J. 2008;25(7):398–402.
8. Jobe J, Ghuysen A, Gerard P, Hartstein G, D'Orio V. Reliability and validity of a new French-language triage algorithm: the ELISA scale. Emerg Med J. 2014;31(2):115–20.
9. Taboulet P, Moreira V, Haas L, Porcher R, Braganca A, Fontaine JP, et al. Triage with the French Emergency Nurses Classification in Hospital scale: reliability and validity. Eur J Emerg Med. 2009;16(2):61–7.
10. Farrokhnia N, Goransson KE. Swedish emergency department triage and interventions for improved patient flows: a national update. Scand J Trauma Resusc Emerg Med. 2011;19:72.
11. Skriver C, Lauritzen MM, Forberg JL, Gaardboe-Poulsen OB, Mogensen CB, Hansen CL, et al. Triage quickens the treatment of the most sick patients. Ugeskr Laeger. 2011;173(40):2490–3.
12. Iversen AK, Kristensen M, Ostervig R, Forberg JL, Schou M, Iversen K. [No evidence that formalized triage is superior to informally structured triage]. Ugeskr Laeger. 2015;177(30):2–6.
13. Johansen MB, Forberg JL. Nurses' evaluation of a new formalized triage system in the emergency department - a qualitative study. Dan Med Bull. 2011;58(10):A4311.
14. Danish Emegency Process Triage, [cited 2016 14/6]. Available from: www.deptriage.dk. Accessed 14 June 2016.
15. Farrohknia N, Castren M, Ehrenberg A, Lind L, Oredsson S, Jonsson H, et al. Emergency department triage scales and their components: a systematic review of the scientific evidence. Scand J Trauma Resusc Emerg Med. 2011;19:42.
16. Cooke MW, Jinks S. Does the Manchester triage system detect the critically ill? J Accid Emerg Med. 1999;16(3):179–81.
17. JMurray M. The Canadian Triage and Acuity Scale: A Canadian perspective on emergency department triage. Emerg Med (Fremantle). 2003;15(1):6–10.
18. Hardern RD. Critical appraisal of papers describing triage systems. Acad Emerg Med. 1999;6(11):1166–71.
19. Eitel DR, Travers DA, Rosenau AM, Gilboy N, Wuerz RC. The emergency severity index triage algorithm version 2 is reliable and valid. Acad Emerg Med. 2003;10(10):1070–80.
20. Tanabe P, Gimbel R, Yarnold PR, Adams JG. The Emergency Severity Index (version 3) 5-level triage system scores predict ED resource consumption. J Emerg Nurs. 2004;30(1):22–9.
21. Barfod C, Lauritzen MM, Danker JK, Soletormos G, Forberg JL, Berlac PA, et al. Abnormal vital signs are strong predictors for intensive care unit admission and in-hospital mortality in adults triaged in the emergency department - a prospective cohort study. Scand J Trauma Resusc Emerg Med. 2012;20:28.
22. Doherty SR, Hore CT, Curran SW. Inpatient mortality as related to triage category in three New South Wales regional base hospitals. Emerg Med (Fremantle). 2003;15(4):334–40.
23. Martins HM, Cuna LM, Freitas P. Is Manchester (MTS) more than a triage system? A study of its association with mortality and admission to a large Portuguese hospital. Emerg Med J. 2009;26(3):183–6.
24. Plesner LL, Iversen AK, Langkjaer S, Nielsen TL, Ostervig R, Warming PE, et al. The formation and design of the TRIAGE study–baseline data on 6005 consecutive patients admitted to hospital from the emergency department. Scand J Trauma Resusc Emerg Med. 2015;23:106.
25. Iversen AK S, Forberg JL, Schou M, Rasmussen LS, Køber L, Iversen K. Comparison of systematic triage with clinical assessment in prediction of short-term mortality. Scand J Trauma Resusc Emerg Med. 2015;23(1):1.
26. The Capital Region of Denmark. Herlev Hospital Homepage [cited 2016 12/7]. Available from: https://www.herlevhospital.dk/. Accessed 14 June 2016.
27. The Capital Region of Denmark. Bispebjerg Hospital Homepage [cited 2016 12/7]. Available from: https://www.bispebjerghospital.dk/. Accessed 14 June 2016.
28. Barfod C, Lauritzen MM, Danker JK, Soletormos G, Berlac PA, Lippert F, et al. The formation and design of the 'Acute Admission Database'- a database including a prospective, observational cohort of 6279 patients triaged in the emergency department in a larger Danish hospital. Scand J Trauma Resusc Emerg Med. 2012;20:29.
29. Blunt I, Bardsley M, Grove A, Clarke A. Classifying emergency 30-day readmissions in England using routine hospital data 2004-2010: what is the scope for reduction? Emerg Med J. 2015;32(1):44–50.
30. Hoot NR, Aronsky D. Systematic review of emergency department crowding: causes, effects, and solutions. Ann Emerg Med. 2008;52(2):126–36.
31. Richardson DB. Increase in patient mortality at 10 days associated with emergency department overcrowding. Med J Aust. 2006;184(5):213–6.
32. Christensen D, Jensen NM, Maaloe R, Rudolph SS, Belhage B, Perrild H. Nurse-administered early warning score system can be used for emergency department triage. Dan Med Bull. 2011;58(6):A4221.
33. Subbe CP, Slater A, Menon D, Gemmell L. Validation of physiological scoring systems in the accident and emergency department. Emerg Med J. 2006; 23(11):841–5.
34. Considine J, LeVasseur SA, Villanueva E. The Australasian Triage Scale: examining emergency department nurses' performance using computer and paper scenarios. Ann Emerg Med. 2004;44(5):516–23.
35. Iversen K, Gotze JP, Dalsgaard M, Nielsen H, Boesgaard S, Bay M, et al. Risk stratification in emergency patients by copeptin. BMC Med. 2014;12:80.
36. Lyngbaek S, Andersson C, Marott JL, Moller DV, Christiansen M, Iversen KK, et al. Soluble urokinase plasminogen activator receptor for risk prediction in patients admitted with acute chest pain. Clin Chem. 2013;59(11):1621–9.
37. Seymour CW, Cooke CR, Wang Z, Kerr KF, Yealy DM, Angus DC, et al. Improving risk classification of critical illness with biomarkers: A simulation study. J Crit Care. 2013;28(5):541–48.
