# Supplementary material for: The Copenhagen Triage Algorithm is non-inferior to a traditional triage algorithm: A cluster-randomized study
Source: PLoS One. 2019 Feb 4;14(2):e0211769. doi: 10.1371/journal.pone.0211769 (PMC6361446; doi:10.1371/journal.pone.0211769)
Supplement: S2 Protocol — (DOCX) [file pone.0211769.s010.docx]

Protocol – The Triage II Trial

**Table of contents**

[Original protocol (In Danish) 3](#_Toc503526478)

[Original Protocol (translated from Danish) 2](#_Toc503526479)0

[Summary of changes to the protocol 25](#_Toc503526484)

**Triage II**

**Kvalitetssikringsprojekt på Herlev Hospital og Bispebjerg Hospital**

**Projektbeskrivelse november 2014**

**Deltagere**

**Kasper Iversen Kardiologisk Afdeling S, Herlev Hospital**

**Morten Schou, Kardiologisk Afdeling S, Herlev Hospital**

**Thomas Høi Hansen, Kardiologisk Afdeling S, Herlev Hospital**

**Lisbet Ravn, Akut Afdelingen, Herlev Hospital**

**Morten Lind, Akutafdelingen, Herlev Hospital**

**Birgitte Nybo Jensen, Akutafdelingen, Bispebjerg Hospital**

**Rasmus Greibe, Akutafdelingen, Bispebjerg Hospital**

**Carsten Pedersen, Akutafdelingen, Bispebjerg Hospital**

**Andre……**

**Baggrund**

Travlhed i akutmodtagelser er et problem verden over. Da ikke alle patienter kan blive tilset af en læge umiddelbart efter ankomst i modtagelserne, anvendes prioriteringsværktøjer i form af triagemodeller. Triagemodeller har til formål at identificere behandlingsbehovet hos den enkelte patient, og sikre at de mest syge behandles først[1].

Behovet for triage eksemplificeres, når antallet af patienter overstiger antallet af behandlende personale. Inden systematiserede triagemodeller blev indført, anvendte personalet alene en klinisk vurdering til at prioritere behandlingsrækkefølgen hos de indkomne patienter. Systematiseret triage foregår typisk ved, at en kombination af patientens primære symptom, samt vitale parametre, scores efter en præspecificeret algoritme. På denne måde opdeles patienterne i kategorier som vha. farver eller tal signalerer, hvor hurtigt de skal tilses, samt hvor tæt de skal observeres[2].

*Historisk perspektiv*

Begrebet ”triage” er afledt af det franske verbum ”trier” og betyder ”sortere”. Den første systematiserede triagemodel blev udviklet på et lokalt hospital i Australien i 1970’erne. Australien var efterfølgende først til at udvikle og indføre en national femtrins triagemodel, ”National Triage Scale”(NTS), i 1993[3,4]. Modellen er i dag modificeret til ”Australian Triage Scale” (ATS)[5], som har dannet grundlag og inspiration for mange efterfølgende triagemodeller[6]. Tre andre femtrins triagemodeller er i dag internationalt accepterede; Canadian Triage and Acuity Scale (CTAS), Manchester Triage System (MTS), Emergency Severity Index (ESI)[7–9] (se tabel 2).

Modellerne er alle mest brugt i deres oprindelsesland, men herudover indført i varierende grad verden over, hvor også en større mængde lokale modeller er i brug[8,10–12].

I Skandinavien var Sverige i slutningen af 1990’erne først til at sætte fokus på triage[13]. To svenske modeller ”Rapid Emergency Triage and Treatment System”(RETTS)[14] samt ”Adaptive Process Triage” (ADAPT)[15] er siden udviklet. Begge modeller anvender proces-triage, hvor triageringen ikke kun medfører en hastegrad, men ydermere giver standardiserede retningslinier for igangsætning af basal behandling og initial diagnostik.

*Triage i Danmark*

I Danmark er systematiseret triage et relativt nyt fænomen, som særligt vandt indpas i årene 2009-2010[1], hvor ADAPT forsøgsvis blev indført i flere danske akutmodtagelser[16,17]. ADAPT er i dag tilpasset danske forhold i form af ”Danish Emergency Process Triage” (DEPT).

DEPT er for nuværende den mest anvendte skala i danske akutmodtagelser [18]. Vi har udført en rundspørge blandt Danske Regioner, der oplyser, at alle 21 fælles akutmodtagelser på nuværende tidspunkt anvender én af fire forskellige triagemodeller, som dog alle bygger på en femtrins proces triagemodel.

*Evidens for Triage*

Flere triageværktøjer har været indført for først senere at blive retrospektivt validerede[8,24–26]. Internationalt er der ingen klar konsensus om, hvilke parametre der skal indgå i en model, og derfor er der afvigelser modellerne i mellem[2]. Triagemodellerne er typisk konstrueret ud fra konsensus fra ekspertpaneler og ikke ud fra opsamlede data[24].

Den retrospektive validering bygger dels på måling af, om triagemodeller kan prædiktere de patienter, der har komplicerede forløb og dels på undersøgelser af interobservatørvariation.

Gruppen af studier, der søger at validere triagemodeller, ser på sammenhængen mellem en patients tildelte triageniveau og forskellige effektmål såsom indlæggelsestid (length of stay, LOS), ressourceforbrug, indlæggelse på intensivafdeling og mortalitet.

Indlæggelsestid i skadestuen er undersøgt for tre skalaer (CTAS, MTS og ESI) [27–33]. Studierne finder alle en association mellem LOS og den tildelte triagekategori. Alle studierne finder lavest LOS hos de mindst akut triagerede. Længst LOS er i størstedelen af studierne relateret til patienter i de midterste triagekategorier[27,29–33], mens i et enkelt studie hos de mest akut triagerede[28].

Ressourceforbrug er undersøgt for skalaerne CTAS, MTS og ESI[27,28,31–36], og er som hovedregel opgjort på basis af et samlet forbrug af konsultationer, diagnostiske tests samt diverse procedurer[27,31–36]. Alle disse studier finder, at det samlede forbrug er stigende og følger graden af det tildelte triageniveau. Et enkelt studie tager alene udgangspunkt i blodanalyser og billeddiagnostisk[28], og finder at antallet af blodanalyser følger triageniveauet med højest forbrug hos de akut triagerede[28]. For billeddiagnostik er tendensen anderledes med højt forbrug hos både de lavest og højest triagerede patientkategorier[28]. Denne tendens genfindes i et andet af studierne, som bemærker, at billeddiagnostik er den hyppigst udførte undersøgelse på lavt triagerede patienter[34].

De undersøgte triagemodeller er vist at kunne prædiktere mortalitet og er undersøgt for størstedelen af triagemodellerne (ATS, CTAS, MTS, ESI og ADAPT)[28,31,33,37–41]. Studierne finder mortalitetsrater i den højeste triagekategori på mellem 12 og 32% og i den laveste på mellem 0 og 0.1%^.^ Forskellene i mortalitetsrater studierne imellem afspejler sandsynligvis forskelle i stikprøve samt i observationstid.

Ligeledes har to studier undersøgt, om indlæggelse på intensiv eller i telemetri kan forudses af den tildelte triagekategori. Begge studier finder, at de patienter der vurderes mest akutte, også har flest forløb på disse afsnit[41,42].

Studier, der undersøger for interobservatørvariation, er hovedsagligt udført ved brug af skriftlige scenarier, hvor flere sygeplejersker udfører triage på samme patientcase[5,44–51]. Herudover er der studier, som måler interobservatørvariation ud fra en retrospektiv ekspertvurdering af en patients tildelte triageniveau[29,31,36,42,52]. Studiernes resultater er meget varierende (Kappa=0.38-0.99).

Kun for en triagemodel er interobservatørvariation undersøgt i et klinisk studie[33]. I dette studie foretager to sygeplejersker simultan triage af samme patient blindet fra hinandens vurdering. I studiet findes K=0,78 (n=386)[33], hvilket svarer til substantiel overensstemmelse ud fra definitionerne foreslået af Landis og Koch[53]. I studiet anvendes triagemodellen ESI, og sygeplejerskerne har deltaget i obligatorisk undervisning i brug af modellen lige op til studiestart[33].

Nyligt har man på Hillerød Hospital i to perioder af 3 måneders varighed indsamlet data på i alt ca. 12.000 patientforløb. Disse data er endnu ikke publicerede, men viser at DEPT er dårligere end både vital parametre og en klinisk vurdering (eyeball triage) til at prædiktere død inden for 30 dage (se bilag 1).

Det er aldrig undersøgt, om indførsel af systematiseret triage reducerer mortalitet eller komplikationer i forbindelse med indlæggelse i forhold til den tidligere anvendte kliniske vurdering og der er aldrig vist om en triageringsform er bedre end en anden.

**Formål**

Formålet med dette kvalitetssikringsprojekt er ud fra eksisterende danske databaser at udvikle en triageform der bedre og enklere kan identificere patienter med henholdsvis lav og høj risiko. Ved at monitorere effekten af den nye triage, vil vi sikre, at den nye triageform er mindst lige så sikker som DEPT triage i forhold til død inden for 30 dage.

**Udvikling af ny triage**

Den ny triage bygger på antagelser baseret på data fra de to databaser indsamlet i akutmodtagelsen på Hillerød Hospital i 2009-2010 og i 2013..

Antagelse 1 er at basale værdier kan identificere patienter i hhv. høj og lav risiko for død.

Antagelse 2 er at en klinisk vurdering er godt til at identificere patienter i hhv. høj og lav risiko for død.

Triagen vil foregå ved at alle patienter bliver triageret i henhold til basale værdier og ud fra disse tildeles en farve fra grøn til rød. Herefter skal triageringssygeplejersken vurdere om patienten skal fastholde den tildelte triagefarve eller triageres et trin op eller ned.

Til den primære triage er udregnet et scoresystem baseret på databaser fra Hillerød. Scoresystemet er udviklet ved hjælp af multipel logistisk regression med bootstrapping (se bilag 2).

**Målepunkter**

Det primære målepunkt død inden for 30 dage

Sekundære målepunkter er

- - Indlæggelse på intensiv
  - Ventetid til behandling
  - Indlæggelsestid
  - Genindlæggelse inden for 30 dage

**Succeskriterier**

Det primære succeskriterie er at den nye enklere triageringsform ikke er dårligere end den eksisterende triage i forhold til død inden for 30 dage

**Styrkeberegning**

Til at vise at den nye triageringsform ikke er dårligere end den gamle skal der bruges noninferiority statistik. Ved et signifikansniveau på 5%, en power på 80%, mortalitet ved DEPT på 4,4% og en deltaværdi/non inferiority margien på 0,5% skal man bruge en stikprøvestørrelse på 41.628. Denne stikprøvestørrelse vil give en styrke på 0,72 til at vise at den nye triage reducerer mortaliteten med 0,5%.

**Tidsplan**

Observationsperioden starter d 1/2 2015. Herlev Hospital starter med at bruge den nye triagemodel og Bisbebjerg Hospital fortsætter med at bruge DEPT triage. Dette fortsætter ind til der er opsamlet data på 25.000 patienter. Herefter byttes så rundt således at Herlev bruger DEPT triage og Bisbebjerg bruger den nye triage. Dette fortsætter fremadrettet. Når der er opsamlet data på yderligere 25.000 patienter vil data blive opgjort. Der indlægges ca 200-250 patienter på de 2 matrikler i døgnet, og det vil således tage 200-250 dage at opnå en sufficient datamængde til kvalitetsvurderingen. Observationsperioden vil således løbe frem til senest medio oktober 2015.

**Dataopsamling**

Triageringsresultater og værdier vil løbende blive registreret på akutjournalen. Denne vil herefter blive kopieret og sendt til Herlev Hospital med henblik på dataindtastning.

Information om primært og sekundære endepunkter indhentes fra centrale registre.

**Godkendelser**

Studiet er et non-intervention kvalitetsudviklingsprojekt og skal derfor ikke anmeldes til Videnskabsetisk Komite (bilag 3). Studiet vil blive anmeldt til datatilsynet. Kvalitetssikringsprojektet vil ligeledes blive registreret på clinicaltrials.gov

**Økonomi**

Kvalitetsudviklingsprojektet vil foregå som en del af den kliniske hverdag, og der vil derfor ikke være særskilte udgifter til indsamling af data.

Der vil være udgifter til indtastning af data (1 fuldtids sekretær i 8 måneder) og til kopiering af triageark (2 halvtidssekretærer i 8 måneder)

Udgifter til disse 2 sekretærstillinger vil blive søgt dækket af Herlev Hospital og Bisbebjerg Hospital.

**Afrapportering**

Når data er indsamlet vil data blive opgjort og afrapporteres umiddelbart til hospitalsledelser på de deltagende hospitaler mhp. valg af fremtidig triageform.

**Referencer**

1. Lindberg SØ, Lerche la Cour J, Folkestad L, Hallas P, Brabrand M. The use of triage in Danish emergency departments. Dan Med Bull [Internet] 2011;58:A4301.Available from: http://www.ncbi.nlm.nih.gov/pubmed/21975149

2. Farrohknia N, Castrén M, Ehrenberg A, Lind L, et al. Emergency department triage scales and their components: a systematic review of the scientific evidence. Scand J Trauma Resusc Emerg Med [Internet] 2011 [cited 2013 Oct 30];19:42.Available from: http://www.pubmedcentral.nih.gov/articlerender.fcgi?artid=3150303&tool=pmcentrez&rendertype=abstract

3. Australasia C of EN. Position Statement – Triage and the Australasian Triage Scale. 2012.

4. Meek R, Phiri W. Australasian Triage Scale: consumer perspective. Emerg Med Australas [Internet] 2005 [cited 2014 Apr 16];17:212–7.Available from: http://www.ncbi.nlm.nih.gov/pubmed/15953221

5. Grouse AI, Bishop RO, Bannon AM. The Manchester Triage System provides good reliability in an Australian emergency department. Emerg Med J [Internet] 2009 [cited 2013 Oct 30];26:484–6.Available from: http://www.ncbi.nlm.nih.gov/pubmed/19546267

6. Fitzgerald G, Jelinek GA, Scott D, Gerdtz MF. Republished paper: Emergency department triage revisited. Postgrad Med J [Internet] 2010 [cited 2014 Apr 8];86:502–8.Available from: http://www.ncbi.nlm.nih.gov/pubmed/20709773

7. Weyrich P, Christ M, Celebi N, Riessen R. Triage systems in the emergency department. Med Klin Intensivmed Notfmed [Internet] 2012 [cited 2014 Mar 7];107:67–78; quiz 79.Available from: http://www.ncbi.nlm.nih.gov/pubmed/22349480

8. Moll HA. Challenges in the validation of triage systems at emergency departments. J Clin Epidemiol [Internet] 2010 [cited 2013 Oct 30];63:384–8.Available from: http://www.ncbi.nlm.nih.gov/pubmed/19875271

9. Christ M, Grossmann F, Winter D, Bingisser R, Platz E. Modern triage in the emergency department. Dtsch Arztebl Int [Internet] 2010 [cited 2014 Mar 7];107:892–8.Available from: http://www.pubmedcentral.nih.gov/articlerender.fcgi?artid=3021905&tool=pmcentrez&rendertype=abstract

10. Chi C-H, Huang C-M. Comparison of the Emergency Severity Index (ESI) and the Taiwan Triage System in predicting resource utilization. J Formos Med Assoc [Internet] Formosan Medical Association & Elsevier; 2006 [cited 2014 Apr 2];105:617–25.Available from: http://www.ncbi.nlm.nih.gov/pubmed/16935762

11. Taboulet P, Moreira V, Haas L, Porcher R, et al. Triage with the French Emergency Nurses Classification in Hospital scale: reliability and validity. Eur J Emerg Med [Internet] 2009 [cited 2014 Jan 22];16:61–7.Available from: http://www.ncbi.nlm.nih.gov/pubmed/19194114

12. Bruijns SR, Wallis LA, Burch VC. A prospective evaluation of the Cape triage score in the emergency department of an urban public hospital in South Africa. Emerg Med J [Internet] 2008 [cited 2014 Mar 23];25:398–402.Available from: http://www.ncbi.nlm.nih.gov/pubmed/18573947

13. Farrokhnia N, Göransson KE. Swedish emergency department triage and interventions for improved patient flows: a national update. Scand J Trauma Resusc Emerg Med [Internet] 2011 [cited 2013 Oct 24];19:72.Available from: http://www.pubmedcentral.nih.gov/articlerender.fcgi?artid=3285084&tool=pmcentrez&rendertype=abstract

14. Widgren BR, Jourak M. Medical Emergency Triage and Treatment System (METTS): a new protocol in primary triage and secondary priority decision in emergency medicine. J Emerg Med [Internet] 2011 [cited 2014 Mar 20];40:623–8.Available from: http://www.ncbi.nlm.nih.gov/pubmed/18930373

15. Barfod C, Lauritzen MMP, Danker JK, Sölétormos G, et al. The formation and design of the “Acute Admission Database”- a database including a prospective, observational cohort of 6279 patients triaged in the emergency department in a larger Danish hospital. Scand J Trauma Resusc Emerg Med [Internet] BioMed Central Ltd; 2012 [cited 2013 Sep 8];20:29.Available from: http://www.pubmedcentral.nih.gov/articlerender.fcgi?artid=3403899&tool=pmcentrez&rendertype=abstract

16. Maya Schröder, Tom Jimmy Hansen HJ. Udfordringer i triagefunktion. [cited 2014 Feb 21];Available from: http://www.dsr.dk/Sygeplejersken/Sider/SY-2010-09-5-1-Akutsygepleje.aspx

17. Johansen MB, Forberg JL. Sygeplejerskers erfaringer med formaliseret triage i en fælles akutmodtagelse. 2011;2485.

18. Skriver C LMFJG-POMCHC et al. Triage medfører hurtigere behandling af de mest syge. Ugeskr Laeger 2011;173:2490–3.

19. Medicine ACFE. GUIDELINES ON THE IMPLEMENTATION OF THE AUSTRALASIAN TRIAGE SCALE IN EMERGENCY DEPARTMENT. 2013 p. 1–8.

20. Bullard MJ, Unger B, Spence J, Grafstein E. Revisions to the Canadian Emergency Department Triage and Acuity Scale (CTAS) adult guidelines. CJEM [Internet] 2008;10:136–51.Available from: http://www.ncbi.nlm.nih.gov/pubmed/18371252

21. Group MT. Emergency Triage: Manchester Triage Group [Internet]. 1996 [cited 2014 Apr 24].Available from: http://books.google.dk/books/about/Emergency_Triage.html?id=xEQIGwAACAAJ&pgis=1

22. Emergency Severity Index - A Triage Tool for Emergency Department Care, Implementation Handbook [Internet]. Agency Healthc Res Qual 2012 [cited 2014 Apr 24].Available from: http://www.ahrq.gov/professionals/systems/hospital/esi/esihandbk.pdf

23. Nordberg M, Lethvall S, Castrén M. The validity of the triage system ADAPT. Scand J Trauma Resusc Emerg Med [Internet] BioMed Central; 2010 [cited 2014 Mar 20];18:P36.Available from: http://www.ncbi.nlm.nih.gov/pmc/articles/PMC2941950/

24. Hardern RD. Critical appraisal of papers describing triage systems. Acad Emerg Med [Internet] 1999 [cited 2013 Oct 30];6:1166–71.Available from: http://www.ncbi.nlm.nih.gov/pubmed/10569391

25. Cooke MW, Jinks S. Does the Manchester triage system detect the critically ill? J Accid Emerg Med [Internet] 1999;16:179–81.Available from: http://www.pubmedcentral.nih.gov/articlerender.fcgi?artid=1343329&tool=pmcentrez&rendertype=abstract

26. J Murray M. The Canadian Triage and Acuity Scale: A Canadian perspective on emergency department triage. Emerg Med (Fremantle) [Internet] 2003 [cited 2014 Mar 31];15:6–10.Available from: http://www.ncbi.nlm.nih.gov/pubmed/12656779

27. Storm-Versloot MN, Ubbink DT, Kappelhof J, Luitse JSK. Comparison of an informally structured triage system, the emergency severity index, and the manchester triage system to distinguish patient priority in the emergency department. Acad Emerg Med [Internet] 2011 [cited 2014 Feb 4];18:822–9.Available from: http://www.ncbi.nlm.nih.gov/pubmed/21843217

28. Jiménez JG, Murray MJ, Beveridge R, Pons JP, et al. Implementation of the Canadian Emergency Department Triage and Acuity Scale (CTAS) in the Principality of Andorra: Can triage parameters serve as emergency department quality indicators? CJEM [Internet] 2003;5:315–22.Available from: http://www.ncbi.nlm.nih.gov/pubmed/17466139

29. Wuerz RC, Travers D, Gilboy N, Eitel DR, Rosenau A, Yazhari R. Implementation and refinement of the emergency severity index. Acad Emerg Med [Internet] 2001;8:170–6.Available from: http://www.ncbi.nlm.nih.gov/pubmed/11157294

30. Grossmann FF, Zumbrunn T, Frauchiger A, Delport K, Bingisser R, Nickel CH. At risk of undertriage? Testing the performance and accuracy of the emergency severity index in older emergency department patients. Ann Emerg Med [Internet] 2012 [cited 2014 Apr 7];60:317–25.e3.Available from: http://www.ncbi.nlm.nih.gov/pubmed/22401951

31. Grossmann FF, Nickel CH, Christ M, Schneider K, Spirig R, Bingisser R. Transporting clinical tools to new settings: cultural adaptation and validation of the Emergency Severity Index in German. Ann Emerg Med [Internet] 2011 [cited 2014 Apr 8];57:257–64.Available from: http://www.ncbi.nlm.nih.gov/pubmed/20952097

32. Tanabe P, Gimbel R, Yarnold PR, Adams JG. The Emergency Severity Index (version 3) 5-level triage system scores predict ED resource consumption. J Emerg Nurs [Internet] 2004 [cited 2014 Mar 21];30:22–9.Available from: http://www.ncbi.nlm.nih.gov/pubmed/14765078

33. Eitel DR, Travers DA, Rosenau AM, Gilboy N, Wuerz RC. The emergency severity index triage algorithm version 2 is reliable and valid. Acad Emerg Med [Internet] 2003 [cited 2014 Mar 10];10:1070–80.Available from: http://www.ncbi.nlm.nih.gov/pubmed/14525740

34. Elshove-Bolk J, Mencl F, van Rijswijck BTF, Simons MP, van Vugt AB. Validation of the Emergency Severity Index (ESI) in self-referred patients in a European emergency department. Emerg Med J [Internet] 2007 [cited 2014 Apr 2];24:170–4.Available from: http://www.pubmedcentral.nih.gov/articlerender.fcgi?artid=2660021&tool=pmcentrez&rendertype=abstract

35. Fernandes CMB, Tanabe P, Gilboy N, Johnson LA, et al. Five-level triage: a report from the ACEP/ENA Five-level Triage Task Force. J Emerg Nurs [Internet] 2005 [cited 2014 Feb 24];31:39–50; quiz 118.Available from: http://www.sciencedirect.com/science/article/pii/S0099176704006919

36. Wuerz RC, Milne LW, Eitel DR, Travers D, Gilboy N. Reliability and validity of a new five-level triage instrument. Acad Emerg Med [Internet] 2000;7:236–42.Available from: http://www.ncbi.nlm.nih.gov/pubmed/10730830

37. Doherty SR, Hore CT, Curran SW. Inpatient mortality as related to triage category in three New South Wales regional base hospitals. Emerg Med (Fremantle) [Internet] 2003;15:334–40.Available from: http://www.ncbi.nlm.nih.gov/pubmed/14631700

38. Dent A, Rofe G, Sansom G. Which triage category patients die in hospital after being admitted through emergency departments? A study in one teaching hospital. Emerg Med Australas [Internet] 1999 [cited 2014 Apr 3];11:68–71.Available from: http://doi.wiley.com/10.1046/j.1442-2026.1999.00016.x

39. Martins HMG, Cuña LMDCD, Freitas P. Is Manchester (MTS) more than a triage system? A study of its association with mortality and admission to a large Portuguese hospital. Emerg Med J [Internet] 2009 [cited 2014 Apr 16];26:183–6.Available from: http://www.ncbi.nlm.nih.gov/pubmed/19234008

40. Wuerz R. Emergency Severity Index Triage Category Is Associated with Six‐month Survival. Acad Emerg Med [Internet] 2001 [cited 2014 Apr 3];8:1999–2002.Available from: http://onlinelibrary.wiley.com/doi/10.1111/j.1553-2712.2001.tb00554.x/abstract

41. Barfod C, Lauritzen MMP, Danker JK, Sölétormos G, et al. Abnormal vital signs are strong predictors for intensive care unit admission and in-hospital mortality in adults triaged in the emergency department - a prospective cohort study. Scand J Trauma Resusc Emerg Med [Internet] 2012 [cited 2014 Mar 6];20:28.Available from: http://www.pubmedcentral.nih.gov/articlerender.fcgi?artid=3384463&tool=pmcentrez&rendertype=abstract

42. Tanabe P, Gimbel R, Yarnold PR, Kyriacou DN, Adams JG. Reliability and validity of scores on The Emergency Severity Index version 3. Acad Emerg Med [Internet] 2004 [cited 2014 Apr 7];11:59–65.Available from: http://www.ncbi.nlm.nih.gov/pubmed/14709429

43. Wuerz R. Emergency Severity Index Triage Category Is Associated with Six‐month Survival. Acad Emerg Med [Internet] 2001 [cited 2014 Apr 2];8:1999–2002.Available from: http://onlinelibrary.wiley.com/doi/10.1111/j.1553-2712.2001.tb00554.x/abstract

44. Gerdtz M, Bucknall T. Influence of task properties and subjectivity on consistency of triage: a simulation study. J Adv Nurs [Internet] 2007 [cited 2014 Apr 8];Available from: http://onlinelibrary.wiley.com/doi/10.1111/j.1365-2648.2007.04192.x/full

45. Considine J, LeVasseur SA, Villanueva E. The Australasian Triage Scale: examining emergency department nurses’ performance using computer and paper scenarios. Ann Emerg Med [Internet] 2004 [cited 2014 Mar 27];44:516–23.Available from: http://www.ncbi.nlm.nih.gov/pubmed/15520712

46. Manos D, Petrie DA, Beveridge RC, Walter S, Ducharme J. Inter-observer agreement using the Canadian Emergency Department Triage and Acuity Scale. CJEM [Internet] 2002 [cited 2013 Oct 30];4:16–22.Available from: http://www.ncbi.nlm.nih.gov/pubmed/17637144

47. Beveridge R, Ducharme J, Janes L, Beaulieu S, Walter S. Reliability of the Canadian emergency department triage and acuity scale: interrater agreement. Ann Emerg Med [Internet] 1999 [cited 2013 Oct 30];34:155–9.Available from: http://www.ncbi.nlm.nih.gov/pubmed/10424915

48. Göransson K, Ehrenberg A, Marklund B, Ehnfors M. Accuracy and concordance of nurses in emergency department triage. Scand J Caring Sci [Internet] 2005;19:432–8.Available from: http://www.ncbi.nlm.nih.gov/pubmed/16324070

49. Dallaire C, Poitras J, Aubin K, Lavoie A, Moore L. Emergency department triage: do experienced nurses agree on triage scores? J Emerg Med [Internet] 2012 [cited 2013 Oct 30];42:736–40.Available from: http://www.ncbi.nlm.nih.gov/pubmed/22209550

50. Van der Wulp I, van Baar ME, Schrijvers a JP. Reliability and validity of the Manchester Triage System in a general emergency department patient population in the Netherlands: results of a simulation study. Emerg Med J [Internet] 2008 [cited 2014 Mar 31];25:431–4.Available from: http://www.ncbi.nlm.nih.gov/pubmed/18573959

51. Göransson KE, von Rosen A. Interrater agreement: a comparison between two emergency department triage scales. Eur J Emerg Med [Internet] 2011 [cited 2013 Oct 30];18:68–72.Available from: http://www.ncbi.nlm.nih.gov/pubmed/20679900

52. Gräff I, Goldschmidt B, Glien P, Bogdanow M, et al. The German Version of the Manchester Triage System and its quality criteria--first assessment of validity and reliability. PLoS One [Internet] 2014 [cited 2014 Apr 14];9:e88995.Available from: http://www.pubmedcentral.nih.gov/articlerender.fcgi?artid=3933424&tool=pmcentrez&rendertype=abstract

53. Landis JR, Koch GG. The measurement of observer agreement for categorical data. Biometrics [Internet] 1977 [cited 2014 Mar 21];33:159–74.Available from: http://www.ncbi.nlm.nih.gov/pubmed/843571

**Bilag 1**

**ROC kurve for**

1. Triage baseret på vitale værdier – new score
2. Dept triage
3. Eyeball triage

Bilag 2

Prædiktionsmodel


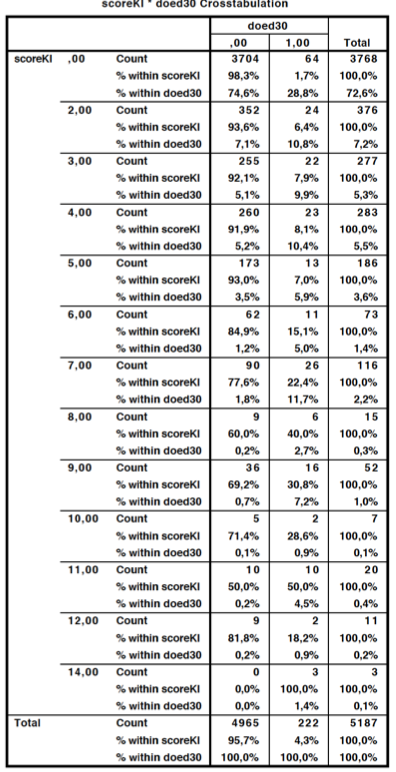


**Study protocol**

**The Triage II trial –**

**key points translated from Danish**

**November 2014**

**Background**

- Crowding in the emergency department is a problem worldwide and has been shown to increase mortality.
- Since the introduction of the first triage system in Australia in the 1970s most countries have implemented triage algorithms in the emergency departments.
- In Denmark, triage was implemented from 2009-2010. The most prevalent system used is called Danish Emergency Process Triage (DEPT) a local adaptation of the Swedish triage system ADAPT.
- The evidence of the positive impact of any triage system is limited.
- Recently 12 000 patient visits at Hillerød Hospital have been registered over two periods of three months. These unpublished data suggests that both vital signs and a clinical assessment are superior in predicting patients at risk of mortality within 30 days.

**Aim**

- First, to develop and validate a new simpler triage algorithm that accurately can identify patients at low risk and at high risk using data from Hillerød Hospital. Second, by monitoring the effect of this new triage algorithms we will ensure that it is at least as safe as DEPT with regard to 30-day mortality.

**Endpoints**

- The primary endpoint is 30-day mortality
- Secondary endpoints are: admission to intensive care unit, waiting time for treatment, length of stay, readmissions within 30 days.

Criteria for success

- The new triage algorithm is non-inferior to the exiting with regard to 30-day mortality.

**Sample size**

- 30-day mortality using DEPT is estimated to 4.4%. Using a significance level of 5%, a power of 80%, and a delta value of 0.5% for non-inferiority a sample size of 41.628 is needed. This will equal a strength of 0.72 to show superiority in reducing mortality by 0.5%.

**Timeline**

- The period of observations begin on February 1, 2015.
- Herlev Hospital starts with the new triage system while Bispebjerg continues using DEPT until about 25.000 patients are registered.
- Based on estimates of patient load for each hospital, the minimum period of observation will last until October, 2015.

**Data**

- Triage levels will be registered in paper-based journals and will subsequently be transferred to an electronic database.
- Data on the primary and secondary endpoints will be obtained using the Danish Patient Registries.

**Approval**

- The trial has been submitted to the local ethics committee who waived the need for approval. Data processing will be registered at the Danish Data Protection Agency for approval. The trial will be registered on [clinicaltrials.gov](http://clinicaltrials.gov).

**Funding**

- The trial will be conduceted as part of the clinical practise at each hospital and will not require additional expenses.
- It is estimated that the trial will need funding for 3 secretaries for data collection from paper-based journals. This funding will be provided by the participating hospitals.

**Appendix**

Appendix Figure 1. **ROC curve for**

1. Triage based on vital signs – new score
2. DEPT triage
3. Eyeball triage (clinical assessment)


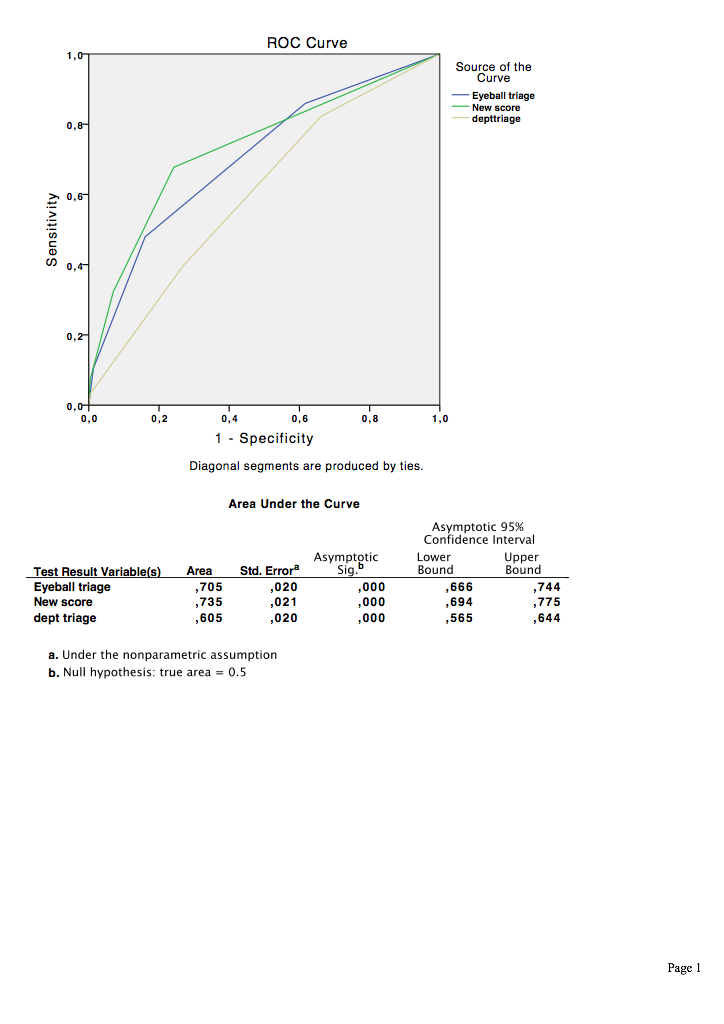


Appendix Figure 2. Prediction model.


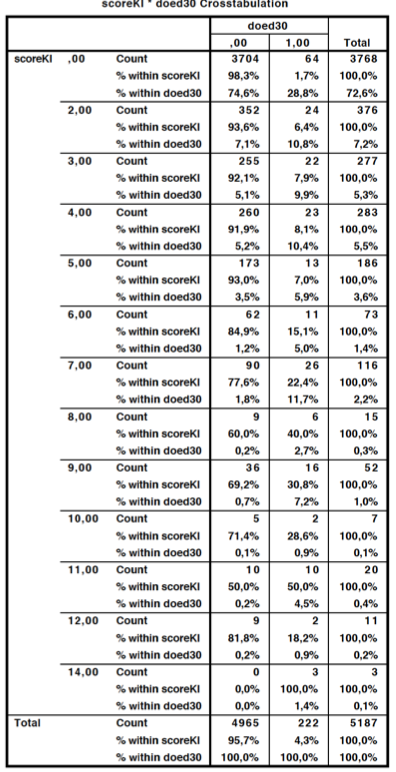


Summary of changes to the protocol

*Changes to the protocol were made before outcome data was available. The final protocol was published as a design article on 10 oktober 2016:*

<https://www.ncbi.nlm.nih.gov/pubmed/27724978>

Upon submission to Clinicaltrials.gov before data was available secondary endpoints were added:

- Acute (48 hour) as well as long-term (90 day) mortality was added as a secondary endpoint as well as readmissions within 90 days to ensure stability of the primary endpoint (30 day mortality) and limit the number of possible patient visits a patient can have in the dataset.
